# Supplementary material for: Nitric Oxide Modulates Metabolic Remodeling in Inflammatory Macrophages through TCA Cycle Regulation and Itaconate Accumulation
Source: Cell Rep. 2019 Jul 2;28(1):218–230.e7. doi: 10.1016/j.celrep.2019.06.018 (PMC6616861; doi:10.1016/j.celrep.2019.06.018)
Supplement: Document S1. Figures S1–S8 and Tables S3 and S4 [file mmc1.pdf]

Cell Reports, Volume *28*

Supplemental Information

Nitric Oxide Modulates Metabolic Remodeling in Inflammatory Macrophages through TCA Cycle Regulation and Itaconate Accumulation

Jade D. Bailey, Marina Diotallevi, Thomas Nicol, Eileen McNeill, Andrew Shaw, Surawee Chuaiphichai, Ashley Hale, Anna Starr, Manasi Nandi, Elena Stylianou, Helen McShane, Simon Davis, Roman Fischer, Benedikt M. Kessler, James McCullagh, Keith M. Channon, and Mark J. Crabtree

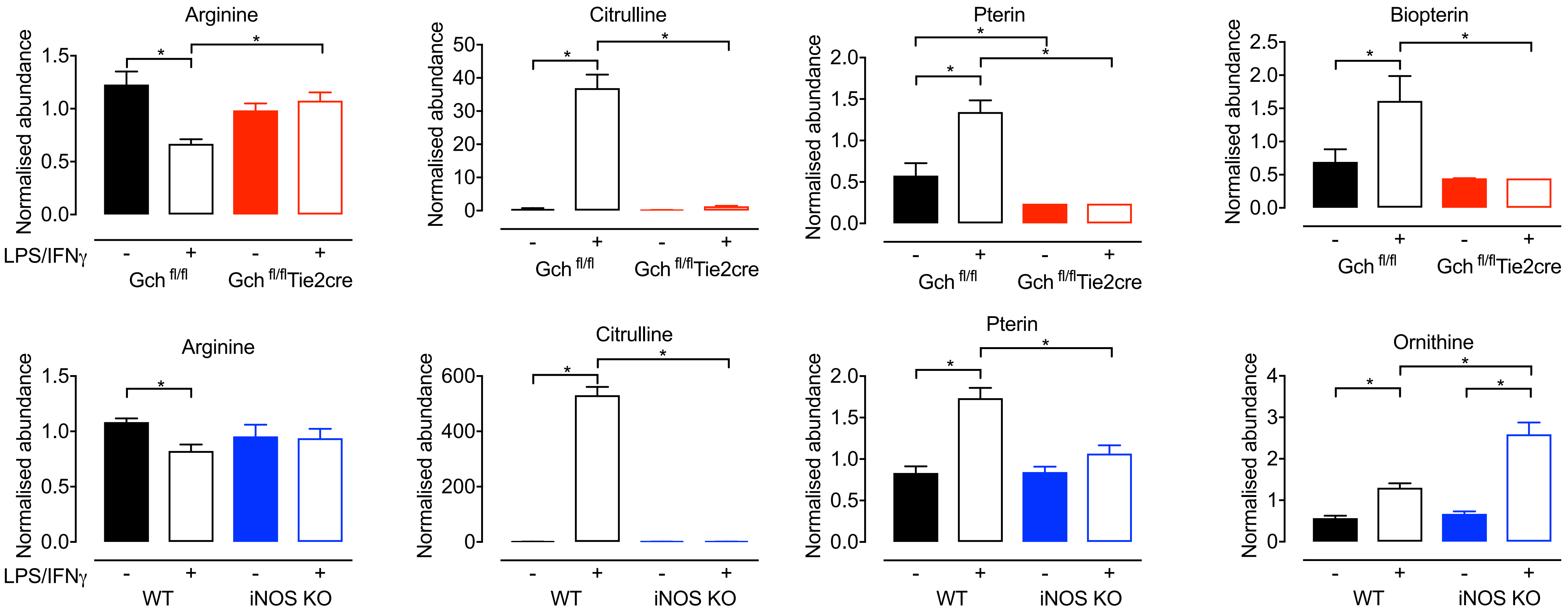

Supplemental figure 1. Quantification of BH4 and NOS related pathway metabolites. Related to Figure 1. Metabolomic measurements of Arginine, Citrulline, Pterin and Biopterin (n=6) Data are mean + SEM. *P* values calculated using 2-way ANOVA with Tukey's post-test (*$P<0.05$).

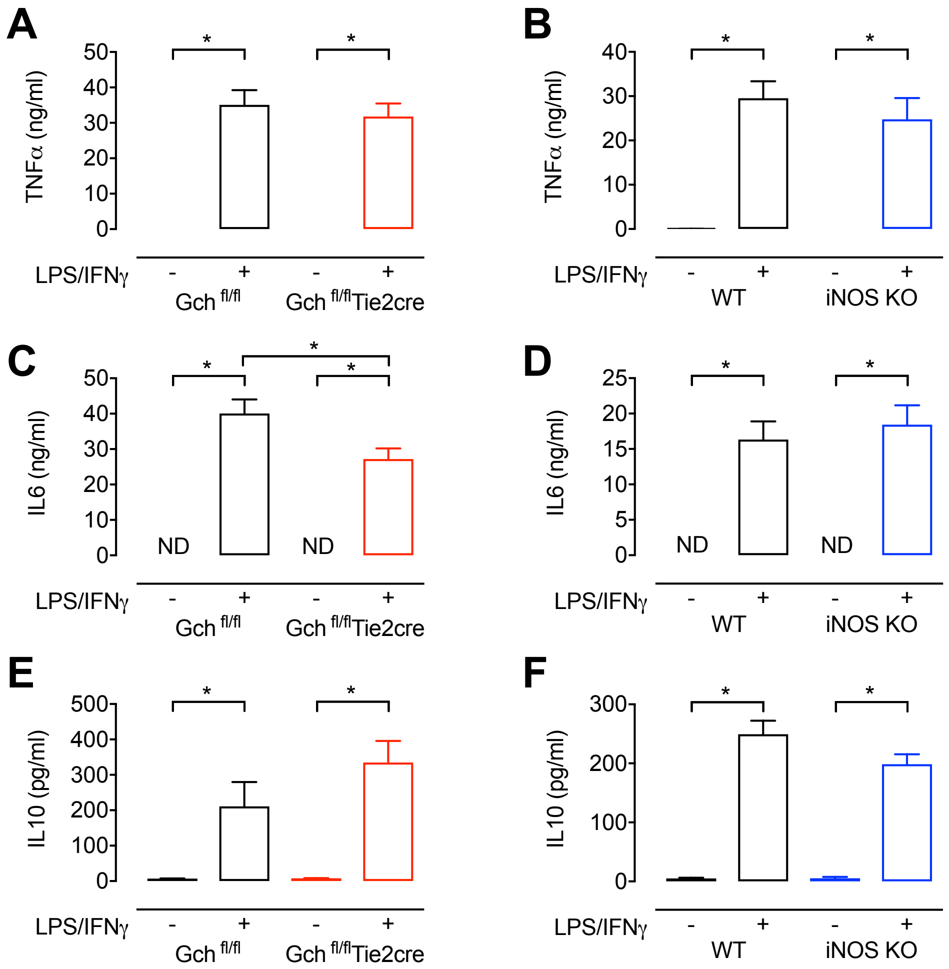

Supplemental figure 2. Inflammatory cytokines are induced in NO-deficient cells. Related to Figure 1. ELISA measurements of TNFα in (A) Gch$^{fl/fl}$Tie2cre and (B) iNOS KO cells, IL-6 in (C) Gch$^{fl/fl}$Tie2cre and (D) iNOS KO cells and IL-10 in (E) Gch$^{fl/fl}$Tie2cre and (F) iNOS KO cells. (n=5) Data are mean + SEM. *P* values calculated using 2-way ANOVA with Tukey's post-test (**P*<0.05).

A

Unstimulated $Gch^{fl/fl}$ Tie2cre vs $Gch^{fl/fl}$

Protein	Fold Change
Squalene synthase	2.69
Adenylate kinase 4, mitochondrial	2.25
Thioredoxin interacting protein	1.59
C5a anaphylatoxin chemotactic receptor 1	1.54
Extracellular serine/threonine protein kinase FAM20C	1.49
PTB-containing, cubilin and LRP1-interacting protein	1.42
Beta-1,4 N-acetylgalactosaminyltransferase 1	1.41
6-phosphofructokinase, liver type	1.37
Class II histocompatibility antigen, M alpha chain	-1.36
C-type lectin domain family 6 member A	-1.36
Urokinase-type plasminogen activator	-1.38
MCG21506	-1.41
Interferon regulatory factor 4	-1.41
Methylcytosine dioxygenase TET2	-1.43
Catenin beta-1	-1.64
Peroxisomal acyl-coenzyme A oxidase 3	-1.93
Placenta-expressed transcript 1 protein	-2.22
Cytochrome P450 2S1	-2.39
Arsenite methyltransferase	-3.76
GTP cyclohydrolase 1 (GTPCH)	-4.40

B

LPS/IFNγ treated $Gch^{fl/fl}$ Tie2cre vs $Gch^{fl/fl}$

Protein	Fold Change
R3H and coiled-coil domain-containing protein 1	4.95
Protein Ms4a7	2.90
Serum amyloid A-3 protein	2.30
NADH dehydrogenase [ubiquinone] 1 alpha subcomplex subunit 12 (NDUFS12)	1.96
Fatty acid desaturase 2	1.89
Protein Gm20547	1.79
Cytochrome P450 4V2	1.77
NADH dehydrogenase [ubiquinone] iron-sulfur protein 6, mitochondrial (NDUFS6)	1.73
Radical S-adenosyl methionine domain-containing protein 2	-1.84
Peroxisomal acyl-coenzyme A oxidase 3	-1.87
DEP domain-containing mTOR-interacting protein	-1.89
Solute carrier family 2, facilitated glucose transporter member 1 (GLUT-1)	-1.97
Platelet-activating factor acetylhydrolase	-1.99
OX-2 membrane glycoprotein	-2.15
Cytochrome P450 2S1	-2.16
Stearoyl-CoA desaturase-3	-2.42
BCL2/adenovirus E1B 19 kDa protein-interacting protein 3	-2.84
Arsenite methyltransferase	-3.07
Isoform 2 of sphingosine kinase 1	-4.67
GTP cyclohydrolase 1 (GTPCH)	-8.96

C

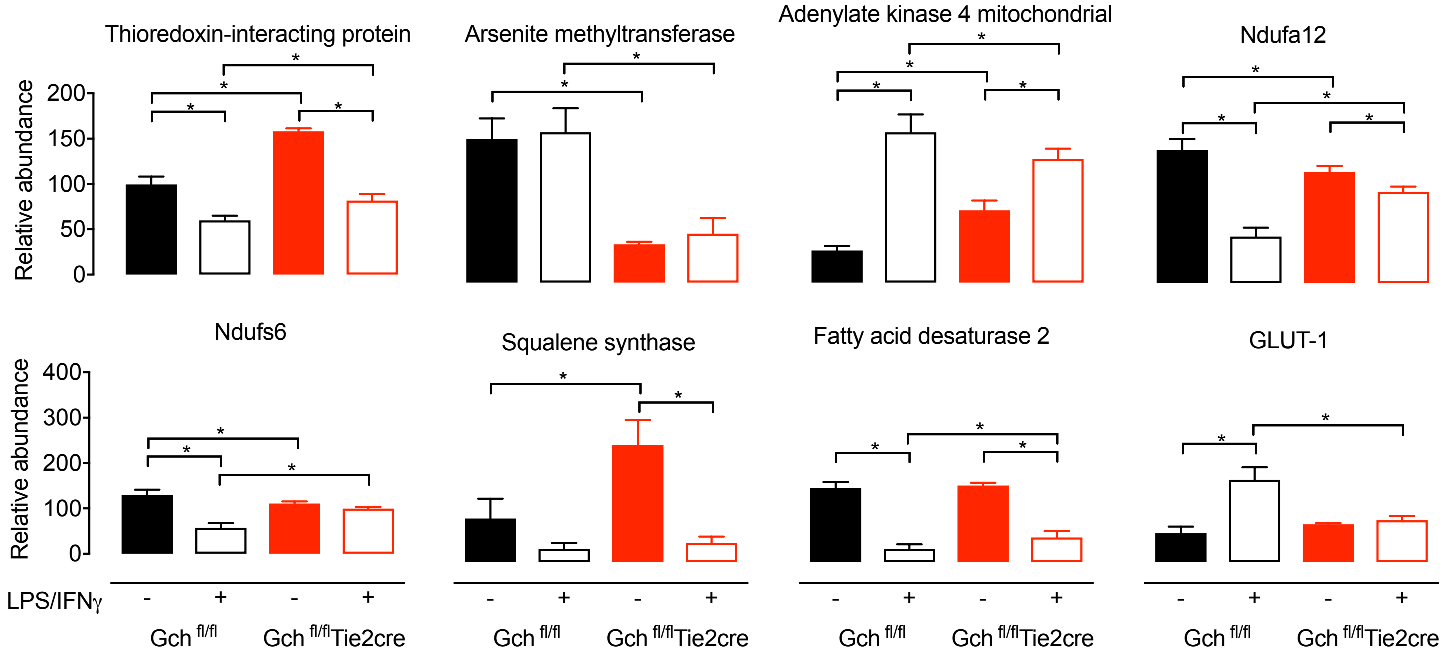

Supplemental figure 3. The proteins with the most changed abundance in $Gch^{fl/fl}$Tie2cre vs $Gch^{fl/fl}$ macrophages. Related to Figure 1. (A-B) The 20 proteins with the most changed abundance in $Gch^{fl/fl}$Tie2cre vs $Gch^{fl/fl}$ macrophages (A) unstimulated or (B) stimulated with LPS and IFNγ. (C) Abundance of selected proteins from A and B. (n=4) Data are mean + SEM (*P<0.05).

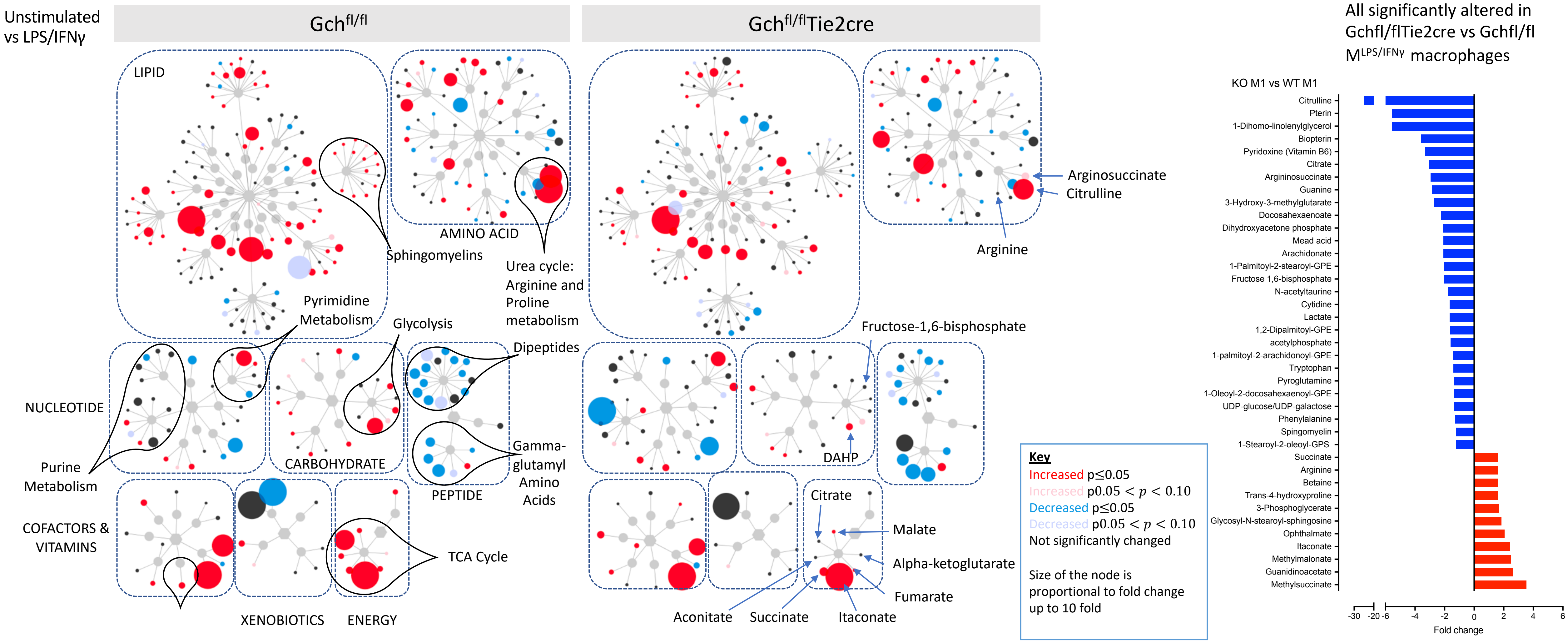

Supplemental figure 4. Cytoscape analysis of metabolomic data from Gch$^{fl/fl}$Tie2cre vs Gch$^{fl/fl}$ macrophages. Related to Figure 4 and 5. (n=6) Data are mean + SEM. *P* values calculated using 2-way ANOVA (*P<0.05).

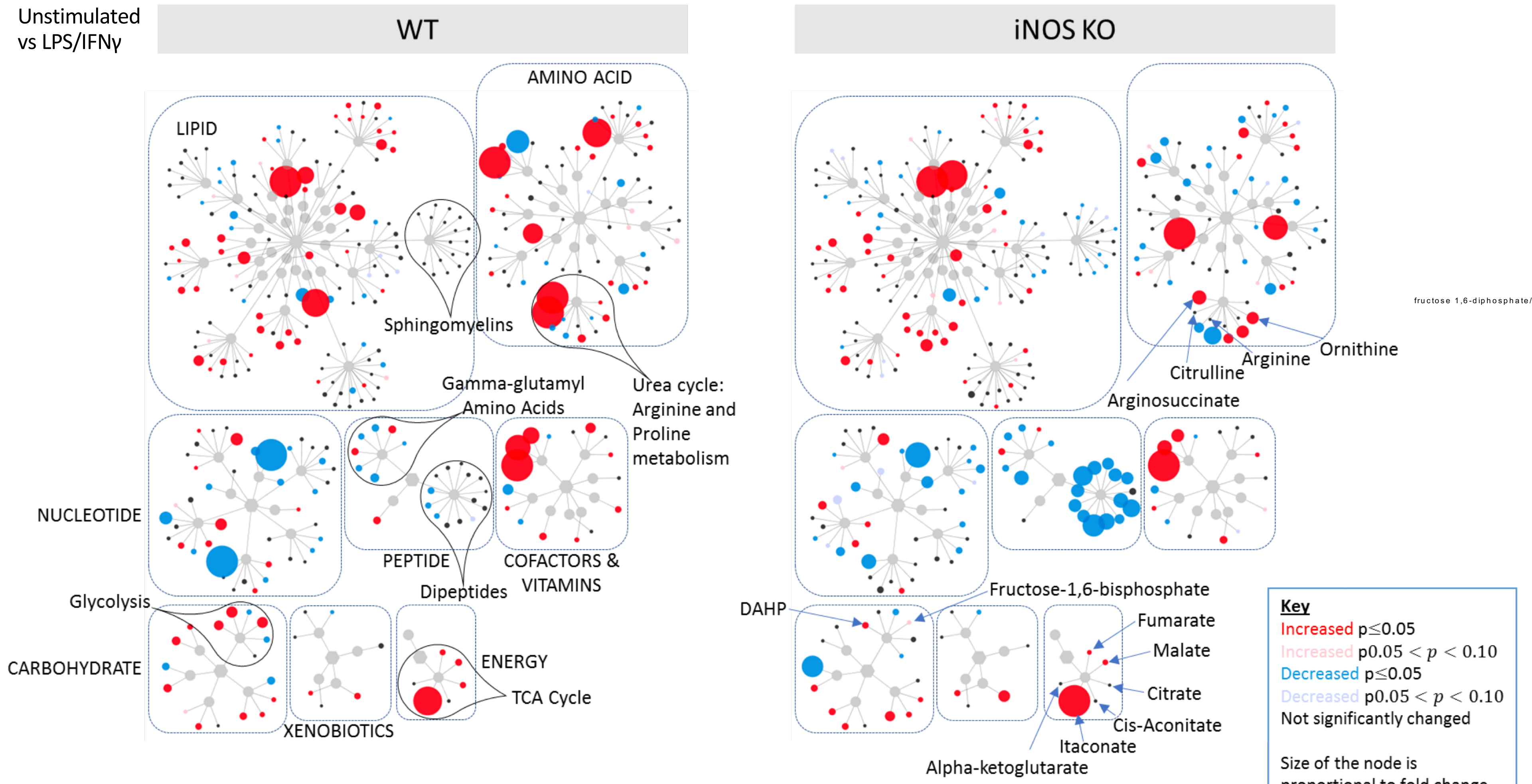

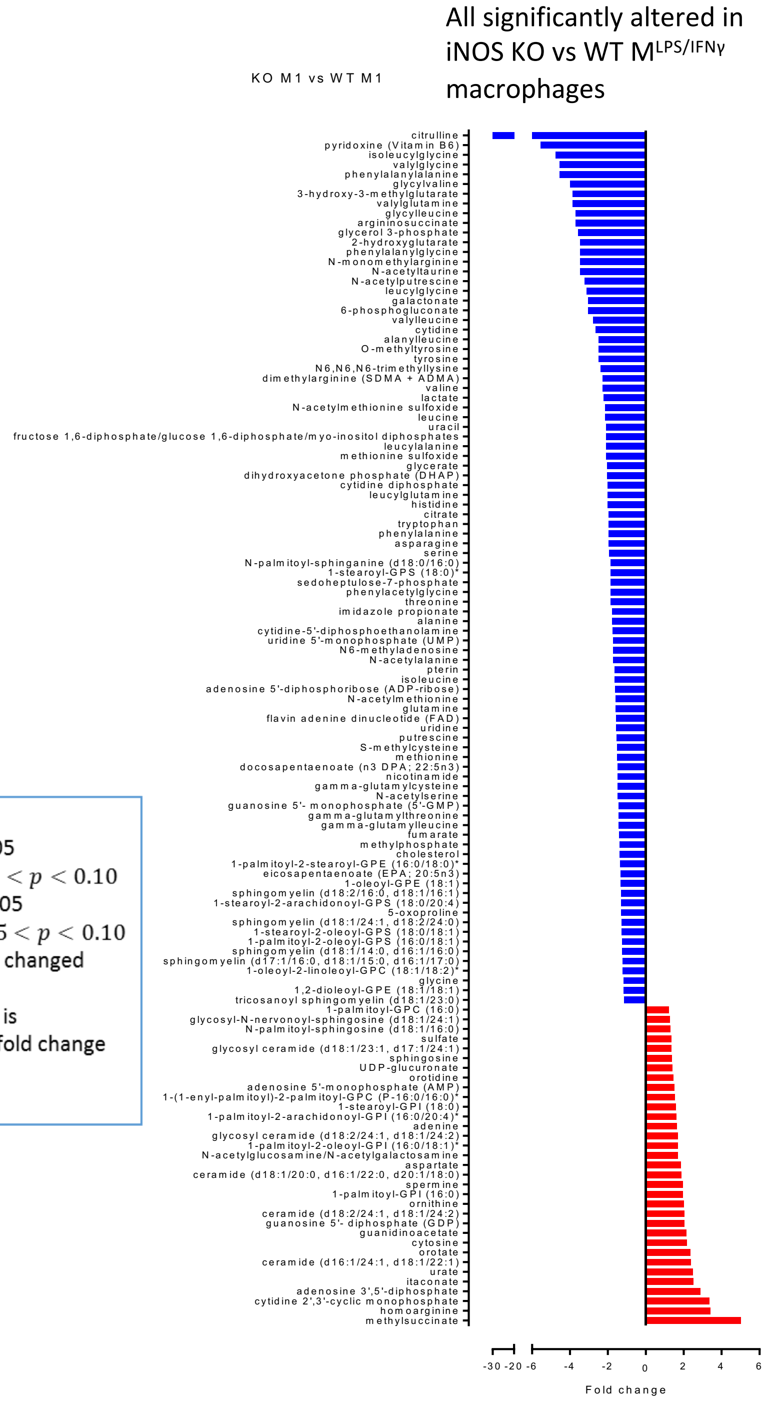

Supplemental figure 5. Cytoscape analysis of metabolomic data from iNOS KO vs WT macrophages. Related to Figure 4 and 5. (n=6) Data are mean + SEM. *P* values calculated using 2-way ANOVA (**P*<0.05).

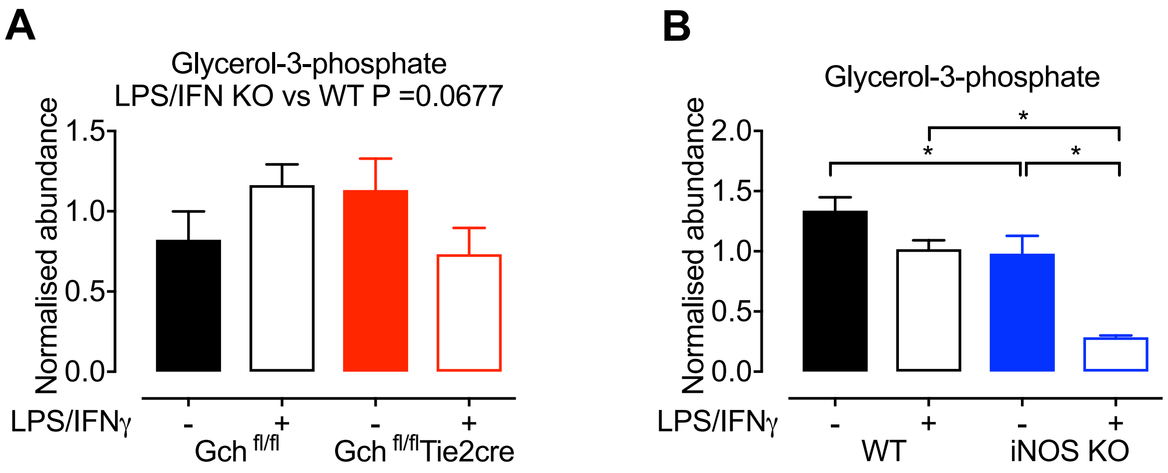

Supplemental figure 6. Glycerol-3-phosphate is decreased in NO deficient activated macrophages. Related to Figure 4. (A-B) Glycerol-3-phosphate abundance measured by metabolomics. (n=6) Data are mean + SEM. *P* values calculated using 2-way ANOVA (**P*<0.05).

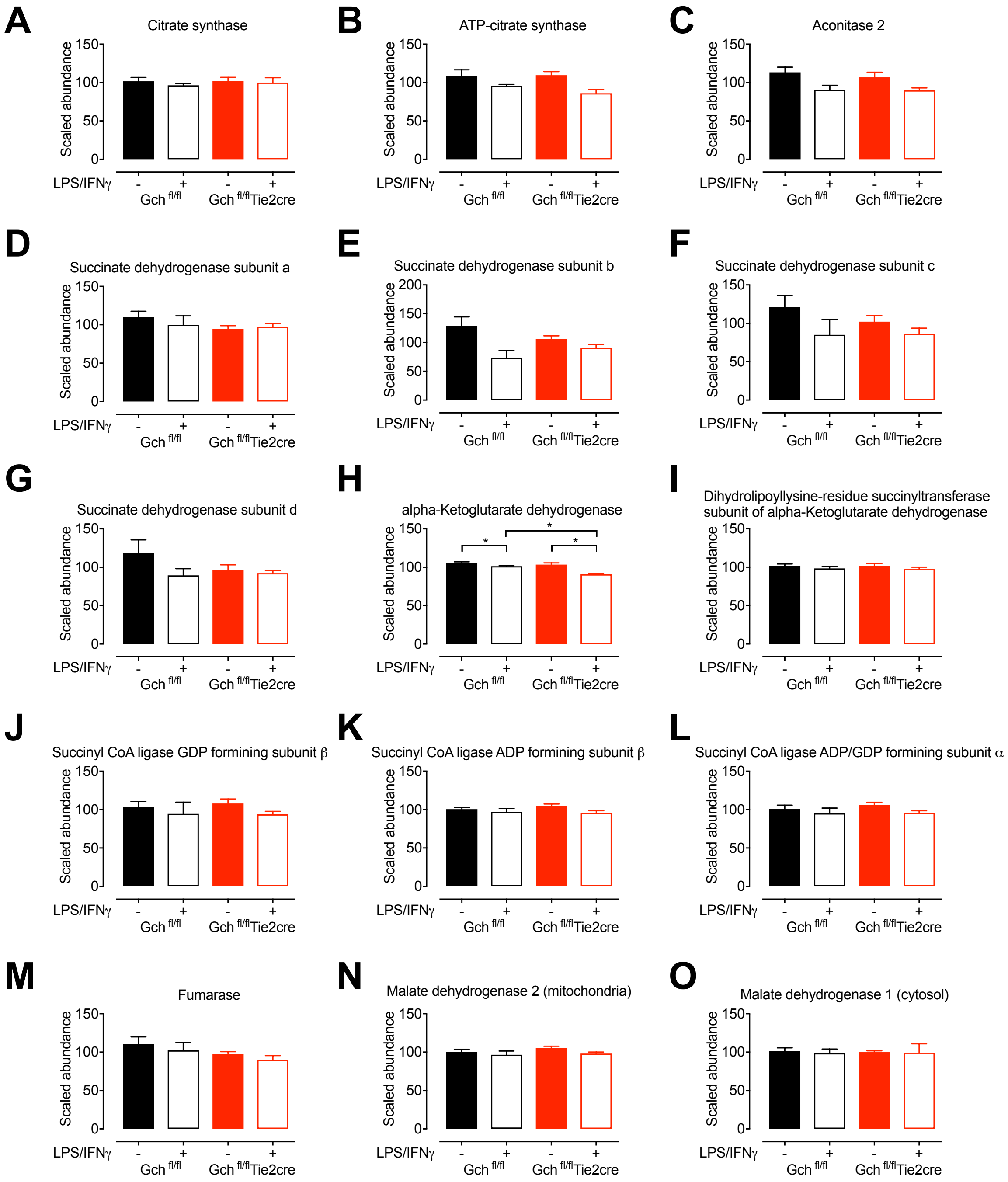

Supplemental figure 7. The abundance of TCA cycle enzymes, measured by metabolomics. Related to Figure 5. (n=6).

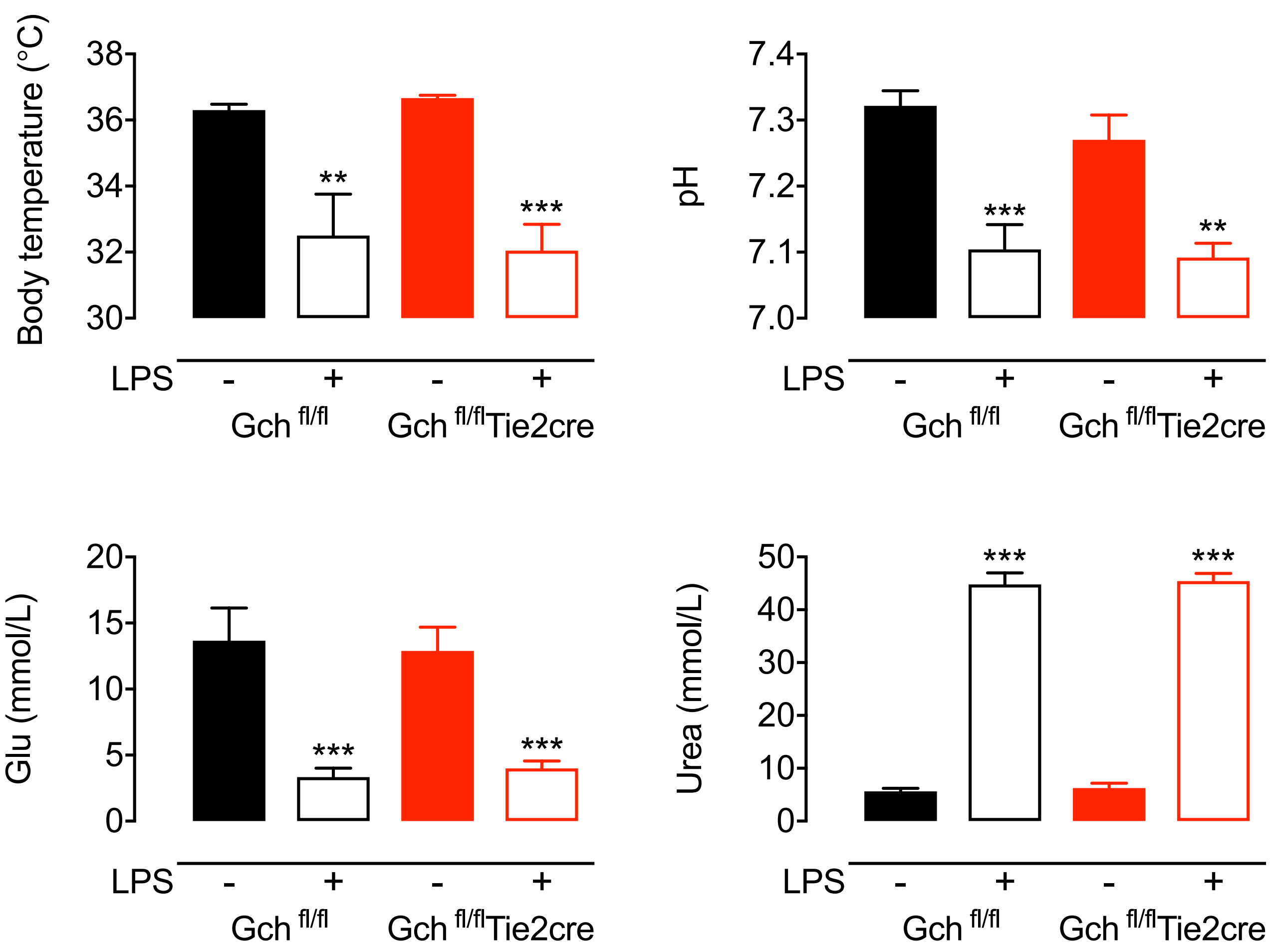

Supplemental figure 8. Physiological responses to acute endotoxaemia, induced by LPS (12.5 mg/kg). Related to Figure 7.

LPS/IFNγ	$Gch^{fl/fl}$		$Gch^{fl/fl}$Tie2cre	
	-	+	-	+
Citrate	0.378 ± 0.158	2.745 ± 0.337	0.804 ± 0.275	0.915 ± 0.238
cis-Aconitate	0.845 ± 0.368	1.770 ± 0.293	1.278 ± 0.378	1.079 ± 0.238
alpha-Ketoglutarate	0.788 ± 0.084	0.973 ± 0.116	1.223 ± 0.163	1.369 ± 0.155
Succinate	0.521 ± 0.080	1.339 ± 0.223	0.677 ± 0.124	2.122 ± 0.193
Fumarate	0.563 ± 0.148	1.160 ± 0.245	1.115 ± 0.283	1.445 ± 0.180
Malate	0.597 ± 0.130	1.132 ± 0.141	0.942 ± 0.183	1.271 ± 0.106
Itaconate	0.118 ± 0.010	2.390 ± 0.483	0.272 ± 080	5.780 ± 0.315

LPS/IFNγ	WT		iNOS KO	
	-	+	-	+
Citrate	0.873 ± 0.069	1.750 ± 0.147	0.865 ± 0.085	0.896 ± 0.098
cis-Aconitate	0.951 ± 0.074	1.095 ± 0.032	0.952 ± 0.078	1.051 ± 0.125
alpha-Ketoglutarate	0.871 ± 0.132	0.902 ± 0.144	0.914 ± 0.066	0.853 ± 0.144
Succinate	ND	ND	ND	ND
Fumarate	0.892 ± 0.090	1.685 ± 0.182	0.781 ± 0.073	1.200 ± 0.108
Malate	0.806 ± 0.082	1.779 ± 0.0260	0.731 ± 0.054	1.324 ± 0.135
Itaconate	0.192 ± 0.049	1.738 ± 0.139	0.234 ± 0.068	4.362 ± 0.232

Supplemental table 3. Metabolite abundances presented in heat map from Figure 5. Metabolite abundance measured by metabolomics. (n=6) Data are mean + SEM.

**Proteins encoded by genes induced by HIF1α**	**References**
GLUT-1	(Chen et al., 2001; Liu et al., 2016; Wang et al., 2017)
Hexokinase-1	(Luo et al., 2006)
Hexokinase-2	(Mathupala et al., 2001; Riddle et al., 2000)
PFKFB3	(Liu et al., 2016; Obach et al., 2004)
Phosphoglucomutase-1	(Hu et al., 2003; Pelletier et al., 2012)
Glycogen synthase	(Pescador et al., 2010)

Supplemental table 4. Glycolytic and glycogen metabolising proteins encoded by genes regulated by HIF1α. Related to Figures 4 and 5.
