# Supplementary material for: Nitric Oxide Modulates Metabolic Remodeling in Inflammatory Macrophages through TCA Cycle Regulation and Itaconate Accumulation
Source: Cell Rep. 2019 Jul 2;28(1):218–230.e7. doi: 10.1016/j.celrep.2019.06.018 (PMC6616861; doi:10.1016/j.celrep.2019.06.018)
Supplement: Document S2. Article plus Supplemental Information [file mmc4.pdf]

Cell Reports

Article

Nitric Oxide Modulates Metabolic Remodeling in Inflammatory Macrophages through TCA Cycle Regulation and Itaconate Accumulation

Graphical Abstract

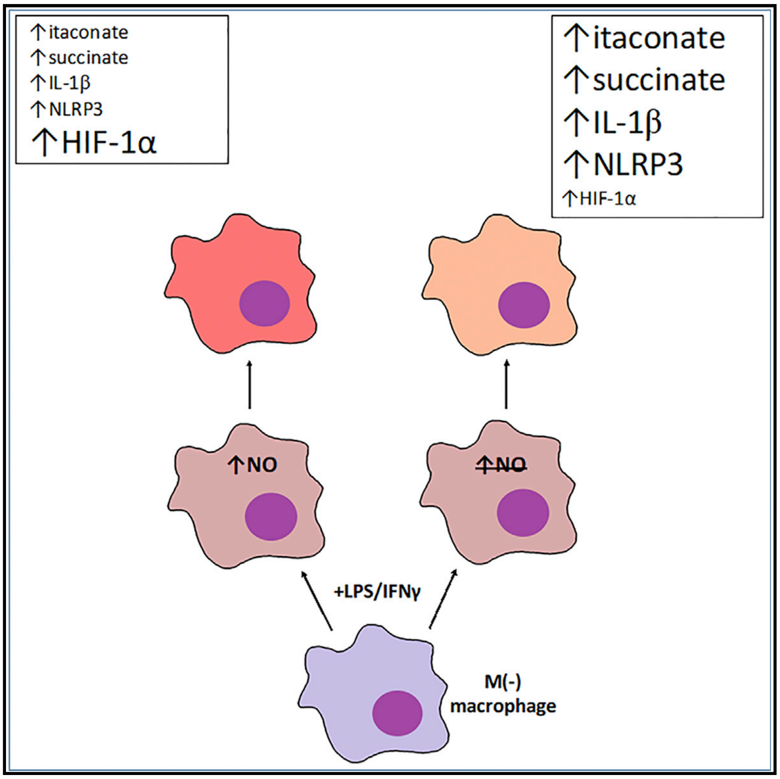

Highlights

- NO orchestrates metabolic remodeling in macrophages responding to LPS+IFNγ
- NO regulates itaconate metabolism in two models of infection and inflammation
- NO determines Complex I subunit abundance in inflammatory macrophages
- Glycolysis is increased in activated NO-deficient cells despite metabolic changes

Authors

Jade D. Bailey, Marina Diotallevi, Thomas Nicol, ..., James McCullagh, Keith M. Channon, Mark J. Crabtree

Correspondence

keith.channon@cardiov.ox.ac.uk (K.M.C.), mark.crabtree@well.ox.ac.uk (M.J.C.)

In Brief

Metabolic remodeling underpins inflammatory macrophage activation, but the modulatory mechanisms are still being elucidated. Bailey et al. show that NO regulates specific changes in the abundance of TCA cycle metabolites, itaconate, and catalytic subunits of Complex I in the respiratory chain in inflammatory murine macrophages both *in vitro* and *in vivo*.

Bailey et al., 2019, Cell Reports *28*, 218–230
July 2, 2019 © 2019 The Author(s).
https://doi.org/10.1016/j.celrep.2019.06.018

OPEN ACCESS
CellPress

Cell Reports
Article

Nitric Oxide Modulates Metabolic Remodeling in Inflammatory Macrophages through TCA Cycle Regulation and Itaconate Accumulation

Jade D. Bailey,[1] Marina Diotallevi,[1] Thomas Nicol,[1] Eileen McNeill,[1] Andrew Shaw,[1] Surawee Chuaiphichai,[1] Ashley Hale,[1] Anna Starr,[2] Manasi Nandi,[2] Elena Stylianou,[3] Helen McShane,[3] Simon Davis,[4] Roman Fischer,[4] Benedikt M. Kessler,[4] James McCullagh,[5] Keith M. Channon,[1,*] and Mark J. Crabtree[1,6,*]
[1]BHF Centre of Research Excellence, Division of Cardiovascular Medicine, Radcliffe Department of Medicine, John Radcliffe Hospital, University of Oxford, Oxford OX3 9DU, UK
[2]School of Cancer and Pharmaceutical Science, Faculty of Life Sciences and Medicine, King's College London, London SE1 9NH, UK
[3]Jenner Institute, University of Oxford, Oxford OX3 7DQ, UK
[4]Target Discovery Institute, Nuffield Department of Medicine, University of Oxford, Roosevelt Drive, Oxford OX3 7FZ, UK
[5]Chemistry Research Laboratory, Department of Chemistry, University of Oxford, Mansfield Road, Oxford OX1 3TA, UK
[6]Lead Contact
*Correspondence: keith.channon@cardiov.ox.ac.uk (K.M.C.), mark.crabtree@well.ox.ac.uk (M.J.C.)
https://doi.org/10.1016/j.celrep.2019.06.018

SUMMARY

Classical activation of macrophages (M(LPS+IFNγ)) elicits the expression of inducible nitric oxide synthase (iNOS), generating large amounts of NO and inhibiting mitochondrial respiration. Upregulation of glycolysis and a disrupted tricarboxylic acid (TCA) cycle underpin this switch to a pro-inflammatory phenotype. We show that the NOS cofactor tetrahydrobiopterin (BH_4) modulates IL-1β production and key aspects of metabolic remodeling in activated murine macrophages via NO production. Using two complementary genetic models, we reveal that NO modulates levels of the essential TCA cycle metabolites citrate and succinate, as well as the inflammatory mediator itaconate. Furthermore, NO regulates macrophage respiratory function via changes in the abundance of critical N-module subunits in Complex I. However, NO-deficient cells can still upregulate glycolysis despite changes in the abundance of glycolytic intermediates and proteins involved in glucose metabolism. Our findings reveal a fundamental role for iNOS-derived NO in regulating metabolic remodeling and cytokine production in the pro-inflammatory macrophage.

INTRODUCTION

Macrophages classically activated by pro-inflammatory stimuli, such as lipopolysaccharide (LPS) and interferon gamma (IFNγ) (M(LPS+IFNγ)), undergo a metabolic switch that includes the upregulation of glycolysis, remodeling of the tricarboxylic acid (TCA) cycle, and the inhibition of mitochondrial respiration (Kelly and O'Neill, 2015). Central to the pro-inflammatory switch is the expression of inducible nitric oxide synthase (iNOS), which generates large quantities of nitric oxide (NO) and requires tetrahydrobiopterin (BH_4) as a cofactor (Tayeh and Marletta, 1989). The NO generated is responsible for inhibiting mitochondrial respiration by nitrosation of NADH dehydrogenase (Complex I) of the electron transport chain (ETC) and reversible inhibition of cytochrome *c* oxidase (Complex IV) (Chouchani et al., 2013; Cleeter et al., 1994; Clementi et al., 1998; Van den Bossche et al., 2016). Consequent upregulation of glycolysis and changes in TCA cycle metabolic intermediates are pivotal determinants of the macrophage inflammatory phenotype. For example, succinate accumulation directs hypoxia-inducible factor 1α (HIF1α)-mediated upregulation of glycolysis and interleukin-1β (IL-1β) production, as well as driving reactive oxygen species (ROS) generation (Mills et al., 2016; Tannahill et al., 2013). Elevated succinate levels have been attributed to the citrate-derived metabolite itaconate inhibiting succinate dehydrogenase (SDH) (Cordes et al., 2016; Lampropoulou et al., 2016). Furthermore, itaconate is an anti-inflammatory mediator that limits levels of inflammatory cytokines and has electrophilic properties that modulate the IκBζ-ATF3 inflammatory axis and NRF2 signaling (Bambouskova et al., 2018; Lampropoulou et al., 2016). Elucidating the mechanisms regulating metabolic pathways and the balance of metabolites is vital for understanding macrophage function, and we set out to investigate the roles of BH_4 and NO in regulating macrophage metabolic remodeling following stimulation with LPS and IFNγ.

The requirement for BH_4 in NOS-derived NO production is well studied; however, we have shown non-cofactor roles for BH_4 in modulating mitochondrial redox signaling in endothelial cells and ROS generation in macrophages (Bailey et al., 2017; McNeill et al., 2015). Furthermore, we recently demonstrated that BH_4-deficient macrophages show enhanced control of mycobacterial infection *in vitro* relative to wild-type cells, whereas iNOS knockout cells showed decreased control (McNeill et al., 2018). This study not only highlighted a NOS-independent effect of BH_4 but also suggested, through transcriptomic analysis, differences in the inflammatory response and cellular metabolism relative to iNOS knockout cells (McNeill et al., 2018). In this study, we therefore used bone-marrow-derived macrophages (BMDMs) and thioglycolate-elicited peritoneal macrophages from two complementary knockout models (*Gch1*, encoding

 © 2019 The Author(s).
This is an open access article under the CC BY license (http://creativecommons.org/licenses/by/4.0/).

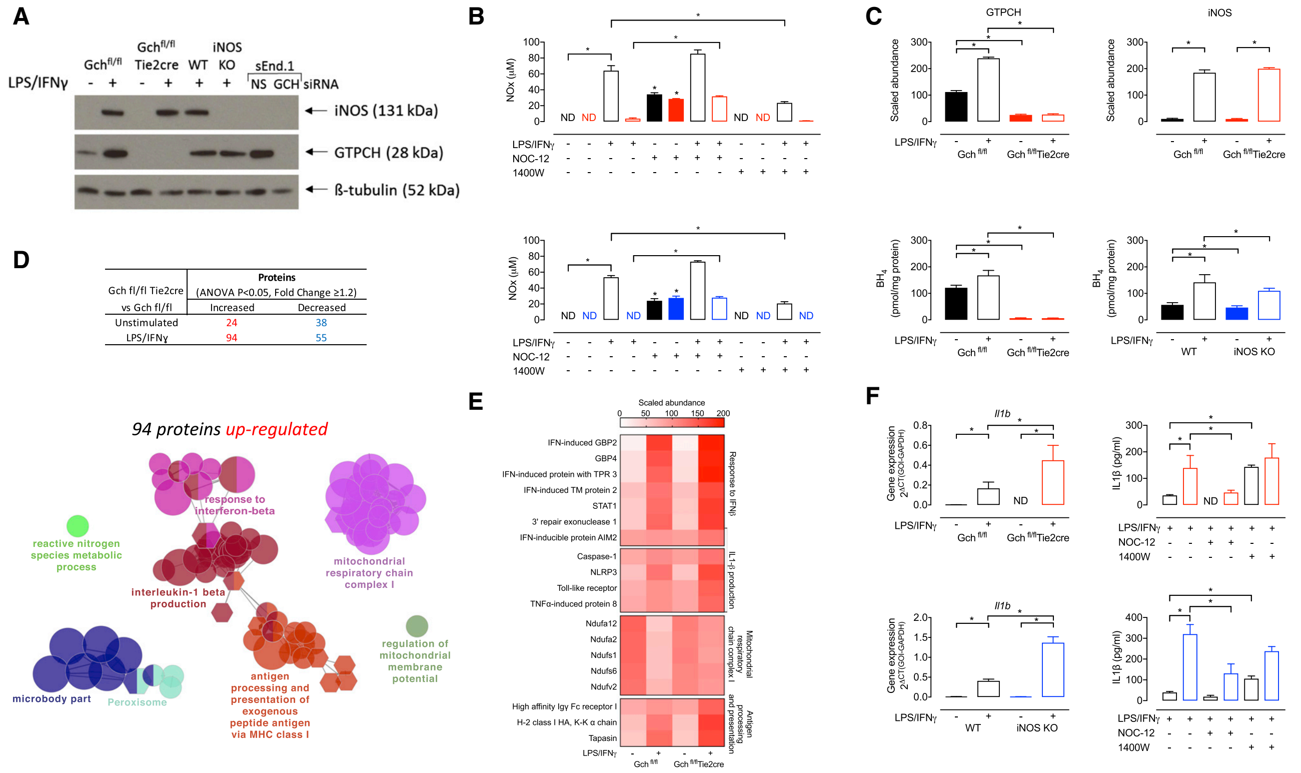

Figure 1. Proteomic Analysis of Upregulated Proteins Highlights BH_4/NO Modulation of Inflammation, Mitochondrial Function, and Metabolism

(A) Western blot analysis of iNOS and GTPCH protein levels in Gch$^{fl/fl}$Tie2cre and iNOS KO BMDMs stimulated with LPS and IFNγ for 16 h. β-Tubulin was used as a loading control. sEnd.1 murine endothelial cells transfected with non-specific (NS) and GCH targeted siRNA were used as positive and negative controls for GTPCH (n = 3).

(B) NO_x (nitrite+nitrate) accumulation in the media measured using an NO analyzer (n = 5–6) (Black bars, Gch$^{fl/fl}$ or WT; Red bars, Gch$^{fl/fl}$Tie2cre; Blue bars, iNOS KO).

(C) The abundance of GTPCH and iNOS proteins were determined by mass spectrometry (n = 4) and intracellular BH_4 quantified using HPLC (n = 5–6).

(D) Number of proteins significantly (ANOVA, $p < 9 \times 10^{-6}$) changed in abundance as determined by mass spectrometric analysis (n = 4) and GO term enrichment analysis of significantly upregulated proteins in Gch$^{fl/fl}$Tie2cre BMDMs stimulated with LPS/IFNγ. The most significant term in each cluster is annotated.

(E) Heatmap showing the scaled abundance of proteins identified in the mostly significantly enriched GO terms from the analysis of upregulated proteins.

(F) *Il1b* gene expression in Gch$^{fl/fl}$Tie2cre and iNOS KO cells measured using qRT-PCR (n = 5) and levels of IL-1β in supernatants from Gch$^{fl/fl}$Tie2cre and iNOS KO cells measured by ELISA (n = 5).

Data are mean + SEM; p values calculated using 2-way ANOVA with Tukey's post-test (*$p < 0.05$).

the BH_4 synthetic enzyme GTP cyclohydrolase [GTPCH], and *Nos2*, encoding iNOS). These cells inherently lacked the capacity to generate inducible NO, and we sought to investigate the effects of NO deficiency on bioenergetic metabolism in activated macrophages while also investigating potential NO-independent effects of BH_4 depletion. We used a multi-"omics" approach followed by systematic validation studies to reveal that BH_4 regulates the abundance of catalytic Complex I subunits, levels of itaconate, TCA cycle and glycolytic metabolites, as well as IL-1β production through NO signaling.

RESULTS

Macrophages Lacking GTPCH or iNOS Do Not Produce NO

To investigate the molecular pathways modulated by NO and BH_4 in M(LPS+IFNγ) macrophages, we used *Gch1*-deficient BMDMs from Gch$^{fl/fl}$Tie2cre mice (McNeill et al., 2015). *Gch1* encodes the protein GTPCH, which is the rate-limiting enzyme in the synthesis of BH_4. As BH_4 is an essential cofactor for iNOS function, resultant BH_4 deficiency leads to macrophages that are unable to produce NO, despite cytokine-induced expression of the iNOS protein (Figures 1A, 1B, and 1C). Due to recent evidence supporting a NOS-independent role for BH_4 in cellular redox signaling (Bailey et al., 2017; McNeill et al., 2015), we also studied *Nos2*$^{-/-}$ (iNOS knockout [KO]) BMDMs. These cells have normal BH_4 levels but are unable to generate NO (Figures 1A, 1B, 1C, and S1) and allow us to identify any NO-independent effects of BH_4 deficiency in macrophage activation. In addition, we have used an NO donor (NOC-12) and iNOS inhibitor (1400W) to further confirm certain NO-dependent affects. NOC-12 was shown to significantly elevate NO_x levels in media from Gch$^{fl/fl}$Tie2cre and iNOS KO M(LPS+IFNγ) macrophages (Figure 1B). Whereas, 1400W significantly decreased NO_x levels

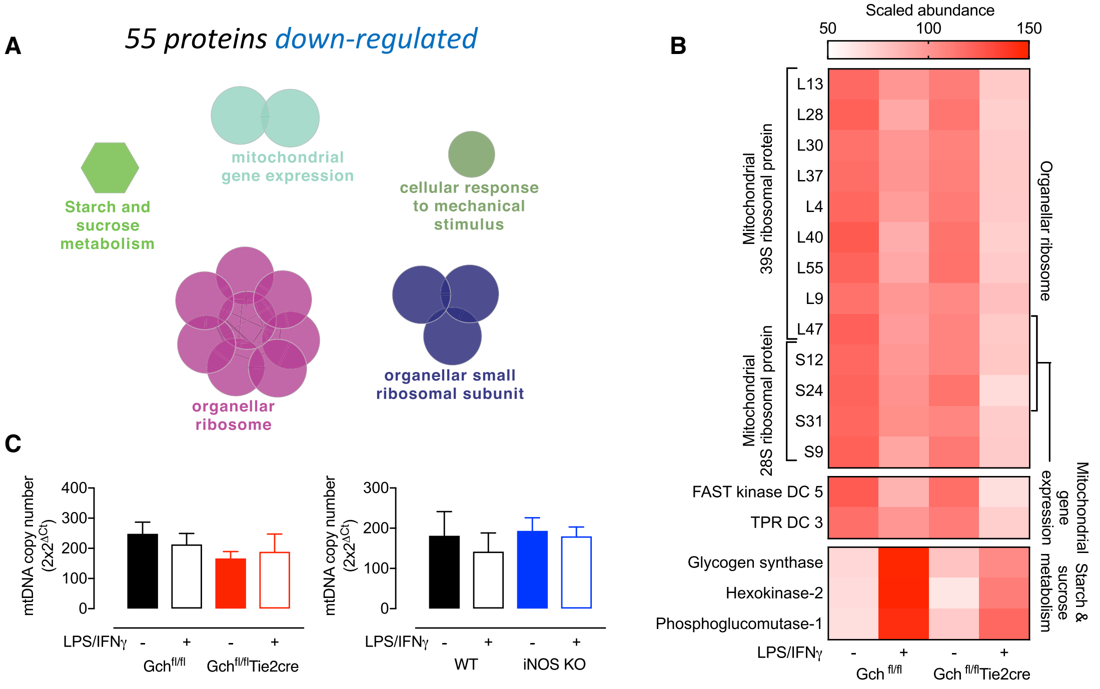

Figure 2. Proteomics Analysis of Downregulated Proteins Highlights BH_4/NO Modulation of Mitochondria and Metabolism

(A) GO term enrichment analysis of significantly downregulated proteins in $Gch^{fl/fl}$Tie2cre BMDMs stimulated with LPS/IFNγ. The most significant term in each cluster is annotated.

(B) Heatmap showing the scaled abundance of proteins identified in the mostly significantly enriched GO terms from the analysis of downregulated proteins (n = 4).

(C) mtDNA content was determined in $Gch^{fl/fl}$Tie2cre and iNOS KO cells (n = 4).

in media from $Gch^{fl/fl}$ and wild-type (WT) M(LPS+IFNγ) cells (Figure 1B).

Proteomic Analysis Reveals BH_4-Mediated Modulation of Inflammation, Mitochondrial Function, and Metabolism

We used tandem mass tag (TMT)-labeling and whole-cell proteomics analysis of $Gch^{fl/fl}$Tie2cre macrophages and $Gch^{fl/fl}$ controls to quantify the relative abundance of 5,704 proteins in unstimulated and LPS/IFNγ-activated cells (PRIDE: PXD010628). We observed the expected changes in GTPCH and iNOS protein abundance (Figures 1A and 1C), demonstrating the validity of the experimental model, and revealed that a select number of proteins differed significantly according to genotype (Figure 1D). Gene Ontology (GO) term enrichment analysis of the 94 proteins that were significantly increased in M(LPS+IFNγ) $Gch^{fl/fl}$Tie2cre versus $Gch^{fl/fl}$ macrophages identified an upregulation of proteins involved in the response to interferon-β (IFN-β), production of IL-1β, antigen presentation, and subunits of mitochondrial respiratory chain Complex I (Figures 1D and 1E). These results suggested both an enhanced response to, and synthesis of, specific inflammatory cytokines in BH_4-deficient macrophages and a potential mechanism linking absence of BH_4 and NO to the maintenance of Complex I function.

To confirm that M(LPS+IFNγ) BH_4-deficient macrophages were producing more of IL-1β, we measured *Il1b* mRNA and IL-1β secretion and found striking elevations in both (Figure 1F). This increased *Il1b* mRNA and IL-1β secretion was also found in M(LPS+IFNγ) iNOS KO cells, indicating that it occurred due to the loss of NO signaling (Figure 1F) and appears to be selective as levels of tumor necrosis factor alpha (TNFα), IL-6, and IL-10 were maintained in NO-deficient cells (apart from a small decrease in IL-6 in stimulated $Gch^{fl/fl}$Tie2cre as previously reported [McNeill et al., 2015]) (Figure S2). The fact that NO regulates IL-1β production was further supported by significantly decreased IL-1β in M(LPS+IFNγ) NO-deficient cells treated with NOC-12 and elevated IL-1β in M(LPS+IFNγ) $Gch^{fl/fl}$ and WT treated with 1400W (Figure 1F).

Enrichment analysis of the 55 decreased proteins highlighted additional mitochondrial and metabolic changes, and showed downregulated proteins involved in the mitochondrial ribosome, mitochondrial gene expression, and carbohydrate metabolism (Figures 2A and 2B). However, analysis of mtDNA content in $Gch^{fl/fl}$Tie2cre and iNOS KO cells indicated no significant changes in mitochondrial content (Figure 2C). Interestingly, metabolic enzymes such as the glucose transporter member 1 (GLUT-1), squalene synthase, fatty acid desaturase-2, and Complex I subunits (Ndufs6 and Ndufa12) were among the 20 most significantly changed proteins (Figure S3). Taken together, these results implicated BH_4 in the modulation of several aspects of metabolism, mitochondrial function, and inflammation, but whether mitochondrial and metabolic changes resulted from loss of NO signaling remained to be confirmed and became the focus of further investigation.

OPEN
ACCESS
CellPress

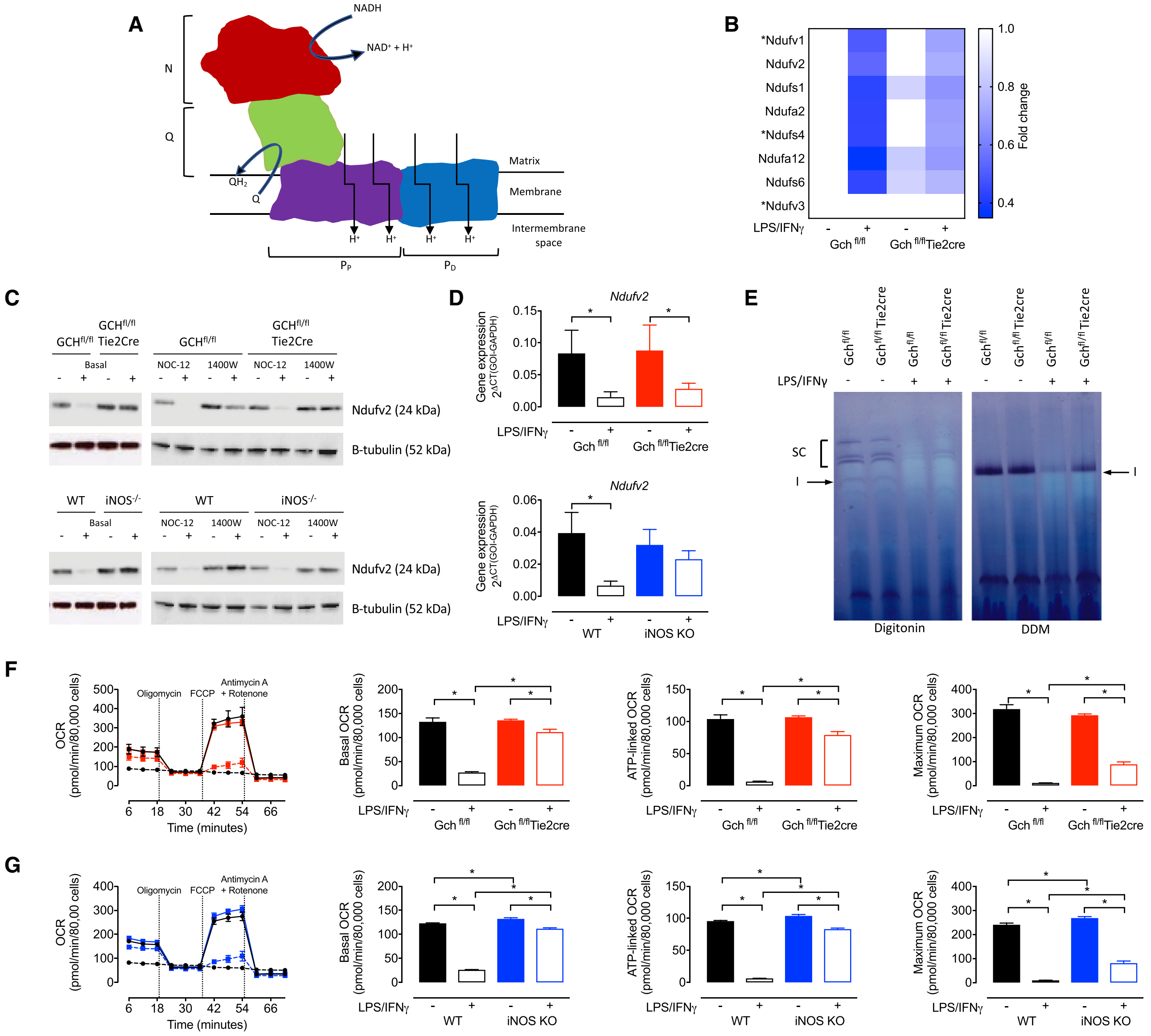

Figure 3. NO-Dependent Inhibition of NADH Dehydrogenase and Mitochondrial Respiration

(A) Structure of the modules (N, Q, P_P, and P_D) making up NADH dehydrogenase (Complex I).

(B) Fold change of N module subunits relative to unstimulated $Gch^{fl/fl}$ cells (n = 4, * denotes subunits not significantly changed by ANOVA).

(C) Ndufv2 protein levels determined by western blotting in $Gch^{fl/fl}$Tie2cre and iNOS KO cells (n = 4).

(D) *Ndufv2* mRNA levels determined by qRT-PCR in $Gch^{fl/fl}$Tie2cre and iNOS KO cells (n = 5).

(E) Complex I (labeled I) in gel activity assay with digitonin to demonstrate supercomplex (SC) abundance and DDM (n-dodecyl-β-D-maltoside) to demonstrate isolated complex I activity in $Gch^{fl/fl}$Tie2cre cells.

(F and G) Oxygen consumption rate (OCR) was measured using $XF^{e}96$ Seahorse bioanalyzer with compounds used to determine basal, ATP-linked and maximum respiration in (F) $Gch^{fl/fl}$Tie2cre (black solid line, unstimulated $Gch^{fl/fl}$; red solid line, unstimulated $Gch^{fl/fl}$Tie2cre; black dashed line, M(LPS+IFNγ) $Gch^{fl/fl}$; red dashed line, M(LPS+IFNγ) $Gch^{fl/fl}$Tie2cre cells) and (G) iNOS KO macrophages (black solid line, unstimulated WT; blue solid line, unstimulated iNOS KO; black dashed line, M(LPS+IFNγ) WT; blue dashed line, M(LPS+IFNγ) iNOS KO cells) (n = 5–6).

Data are mean + SEM; p values calculated using 2-way ANOVA with Tukey's post-test (*$p < 0.05$).

NO Regulates Complex I Function and Mitochondrial Respiration

One of the most striking changes highlighted by the proteomic analysis were changes in several subunits of Complex I, an intricate protein of almost 1,000 kDa, comprising 44 subunits (Guerrero-Castillo et al., 2017). Situated in the mitochondrial inner membrane, Complex I is formed of 4 distinct modules (Figure 3A), and our data revealed significant co-ordinated decreases in 5 out of 8 subunits making up the catalytic NADH binding module (N-module) in stimulated $Gch^{fl/fl}$ cells, with

another 2 subunits (Ndufs4 and Ndufv1) showing a similar pattern but they were not statistically significant (Figure 3B). Upon activation, these decreases were largely attenuated by the absence of BH_4 and NO in $Gch^{fl/fl}$Tie2cre cells—including in the 3 subunits (Ndufv1, Ndufv2, and Ndufs1) thought to be essential for catalysis. Western blot analysis of Ndufv2 in $Gch^{fl/fl}$ and WT cells confirmed depletion of this protein upon stimulation, and the fact that both $Gch^{fl/fl}$Tie2cre and iNOS KO M(LPS+IFNγ) cells retained much greater levels indicated that this was driven by NO (Figure 3C). Furthermore, treatment of $Gch^{fl/fl}$Tie2cre and iNOS KO M(LPS+IFNγ) cells with NOC-12 led to substantially decreased Ndufv2 protein, whereas $Gch^{fl/fl}$ and WT M(LPS+IFNγ) cells treated with 1400W retained Ndufv2 (Figure 3C). Significantly reduced expression of *Ndufv2* in stimulated $Gch^{fl/fl}$ and WT cells suggested that changes in protein levels may result from NO-dependent transcriptional regulation, and restored gene expression in iNOS KO would support this hypothesis (Figure 3D). However, gene expression in M(LPS+IFNγ) $Gch^{fl/fl}$Tie2cre cells was repressed, suggesting involvement of other mechanisms and highlighting a difference between BH_4-replete and -deficient cells. An in-gel activity assay confirmed that Complex I activity was decreased in $Gch^{fl/fl}$ M(LPS+IFNγ) cells but maintained in $Gch^{fl/fl}$Tie2cre M(LPS+IFNγ) cells and also showed that stimulated $Gch^{fl/fl}$ cells had a reduction in Complex I-containing supercomplexes (Figure 3E). These differences were paralleled by changes in oxygen consumption rate (OCR), as basal and ATP-linked respiration were largely maintained in activated cells unable to produce NO in $Gch^{fl/fl}$Tie2cre and iNOS KO, in contrast to the well-established NO-induced inhibition of respiration in $Gch^{fl/fl}$ and WT cells (Figures 3F and 3G). Although maximum respiration was also significantly higher in stimulated NO-deficient cells, this remained no higher than basal levels and significantly lower than unstimulated cells, indicating changes in respiratory capacity that occur after stimulation but by NO-independent mechanisms. These results concur with previous reports that NO modulates Complex I activity and the metabolic switch away from oxidative metabolism in activated macrophages and additionally demonstrates a fundamental and important role for NO in modulating Complex I subunit abundance.

Glycolytic Rate Is Unchanged in the Absence of NO despite Changes in the Abundance of Glycolytic Metabolites and Proteins

The switch from oxidative phosphorylation to glycolysis-mediated ATP synthesis is a well-established characteristic of M(LPS+IFNγ) macrophages (Kelly and O'Neill, 2015). It is directed by increased levels of key proteins under the transcriptional control of HIF1α, including GLUT-1, hexokinase-2, and 6-phosphofructo-2-kinase/fructose-2,6-bisphosphatase 3 (PFKFB3) (Liu et al., 2016; Wang et al., 2017). We observed these same changes in M(LPS+IFNγ) WT macrophages that produce NO (Figures 4A and B). However, in M(LPS+IFNγ) $Gch^{fl/fl}$Tie2cre macrophages, GO term enrichment analysis highlighted comparatively fewer of the enzymes related to "starch and sucrose metabolism," including glycolytic enzyme hexokinase-2 and proteins directing glycogen storage and release (Figures 2A and 2B). Furthermore, a refined search of the data from $Gch^{fl/fl}$Tie2cre macrophages revealed changes in several other proteins involved in glucose metabolism and glycolysis (Figures 4A and 4B), including significant decreases in GLUT-1, hexokinase-1, and PFKFB3, along with increases in hexokinase-3, triose phosphate isomerase, phosphoglycerate kinase, and glycerol-catabolising enzymes (Figures 4A and 4B). To explore the consequences of these changes in protein abundance and whether they were dependent on changes in NO signaling, we used mass spectrometry to measure metabolites in macrophages from $Gch^{fl/fl}$Tie2cre and iNOS KO mice (Figures S4 and S5; Tables S1 and S2). We found marked changes in the abundance of certain glycolytic intermediates in NO-deficient versus -replete M(LPS+IFNγ) cells (Figures 4C and 4D), as well as the glycerol metabolite glycerol-3-phosphate that feeds into this pathway (Table S3). The relative changes in metabolite abundance were very similar in stimulated $Gch^{fl/fl}$ versus $Gch^{fl/fl}$Tie2cre and WT versus iNOS KO cells, indicating that these BH_4-induced changes resulted from the loss of NO. In earlier parts of the glycolysis pathway, there was less fructose-1,6-bisphosphate and dihyroxyacetone-phosphate in NO-deficient cells; however, in later steps there was a small increase of 3-phosphoglycerate (only in $Gch^{fl/fl}$Tie2cre) and no difference in levels of the later products of glycolysis, phosphoenolpyruvate, and pyruvate (Figures 4E and S6).

The product of glycolysis, pyruvate, is converted into lactate or oxidized in the TCA cycle, and both lead to acidification of the media through proton extrusion, which can be used to estimate glycolytic activity. Intracellular lactate was increased in NO-replete M(LPS+IFNγ) cells but to a lesser extent in the absence of NO (Figures 4C, 4D, and 4E), and this was similarly observed for extracellular lactate accumulation (Figure 4F). Interestingly, the extracellular acidification rate (ECAR) resulting from glucose oxidation was elevated in all M(LPS+IFNγ) cells regardless of BH_4 or NO levels (Figure 4G). To investigate the proportional effects of lactate versus TCA acidification, the glycolytic proton efflux rate (glycoPER) and percentage PER attributed to glycolysis were calculated in $Gch^{fl/fl}$ versus $Gch^{fl/fl}$Tie2cre cells (Figure 4H). This confirmed that glycoPER—and thus glycolytic rate—was equally elevated in NO-synthesizing and NO-deficient M(LPS+IFNγ) cells and that glycolysis was the major contributor to PER at $> 90\%$ in M(LPS+IFNγ) cells, which was significantly higher than in non-stimulated cells (Figure 4H). In addition, glucose uptake was similarly elevated in M(LPS+IFNγ) macrophages of all genotypes (Figure 4I).

To validate proteomic changes observed in M(LPS+IFNγ) $Gch^{fl/fl}$Tie2cre cells and their dependence on NO, we measured expression of the gene encoding GLUT-1 (*Slc2a1*) and the amount of hexokinase-2 protein in $Gch^{fl/fl}$Tie2cre and iNOS KO cells. This showed a failure to induce transcription of *Slc2a1* (Figure 4J) and decreased levels of hexokinase-2 protein (Figure 4K) in both $Gch^{fl/fl}$Tie2cre and iNOS KO cells. Interestingly, several proteins of lower abundance in activated cells unable to produce BH_4 and NO were those that were previously reported to be transcriptionally regulated by HIF1α (Table S4). We therefore used western blot analysis to determine whether previously reported LPS-stimulated HIF1α stabilization was disrupted in the absence of BH_4 and NO and found a lower abundance of HIF1α in $Gch^{fl/fl}$Tie2cre cells (Figure 4K). HIF1α was like-wise decreased

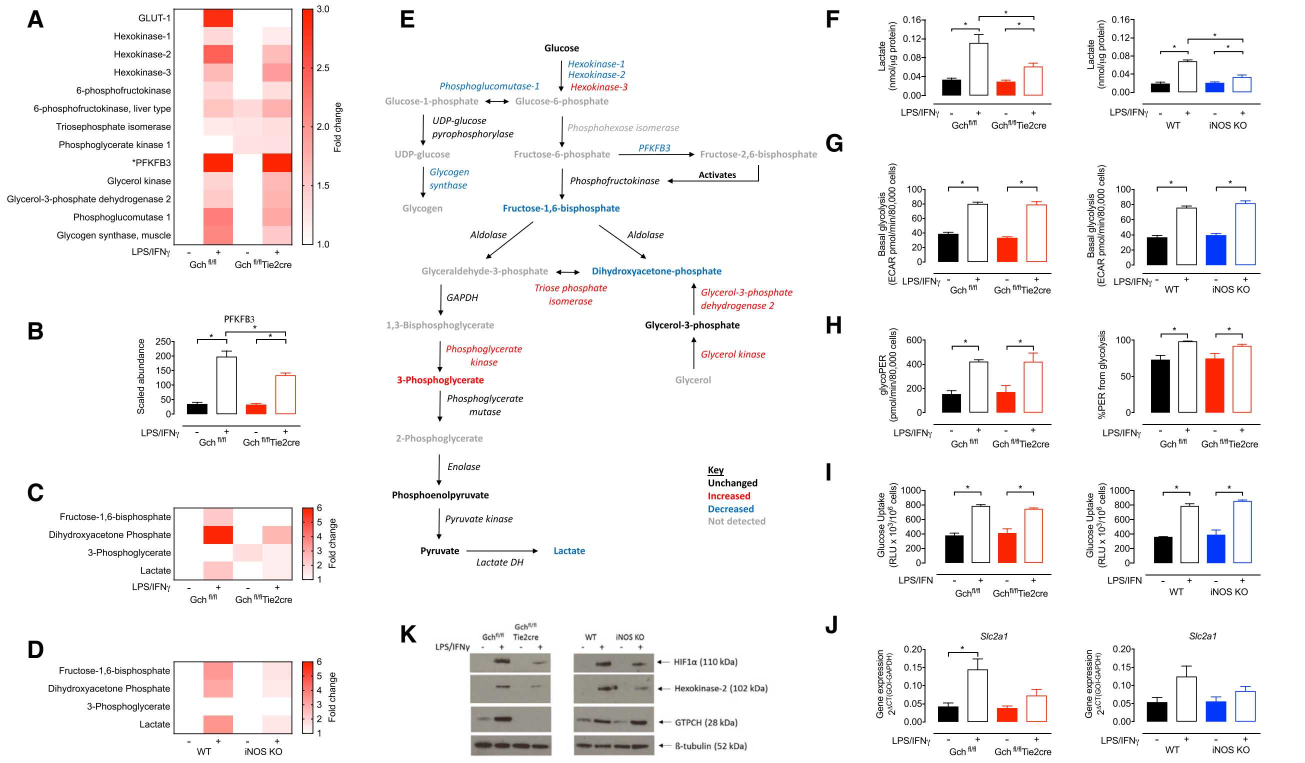

Figure 4. Glycolytic Rate Is Unaffected by Loss of NO Signaling Despite Changes in the Levels of Glycolytic Proteins and Metabolites

(A) Heatmap showing fold changes in abundance of enzymes significantly changed relative to Gch$^{fl/fl}$ unstimulated cells (n = 4, p < 0.05, *PFKB3 = fold change > 3).

(B) Abundance of PFKFB3 protein (n = 4).

(C and D) (C) Fold change heatmaps of significantly changed (p > 0.05) metabolites relative to unstimulated Gch$^{fl/fl}$ or (D) unstimulated WT cells (n = 6).

(E) Glucose metabolism pathway representing changes (>20%) in abundance of enzymes and metabolites in LPS/IFNγ stimulated Gch$^{fl/fl}$Tie2cre versus Gch$^{fl/fl}$ macrophages.

(F) Lactate accumulation measured in medium supernatants from macrophages following overnight stimulation (n = 4).

(G) Basal glycolysis determined using 2-deoxyglucose inhibitable ECAR measured using XF^{e}96 Seahorse bioanalyzer in Gch$^{fl/fl}$Tie2cre and iNOS KO cells (n = 6).

(H) Basal glycolytic rate determined in Gch$^{fl/fl}$Tie2cre cells by calculating glycolytic proton efflux rate (glycoPER) (n = 3).

(H) The contribution of glycolysis to acidification of media in Gch$^{fl/fl}$Tie2cre cells (n = 3).

(I) Measurement of glucose uptake in Gch$^{fl/fl}$Tie2cre and iNOS KO cells (n = 4).

(J) qRT-PCR mRNA measurement of *Slc2a1* gene encoding GLUT-1 protein in Gch$^{fl/fl}$Tie2cre and iNOS KO cells (n = 5).

(K) Western blot analysis of HIF1α and hexokinase 2 proteins in Gch$^{fl/fl}$Tie2cre and iNOS KO cells (n = 4).

Data are mean + SEM; p values calculated using 2-way ANOVA with Tukey's post-test (*p < 0.05).

in iNOS KO cells and implicated loss of NO signaling (Figure 4K). Overall glycolysis is sufficiently upregulated in M(LPS+IFNγ) cells irrespective of BH_4 or NO levels, despite changes in levels of several glycolytic metabolites and proteins involved in glucose metabolism.

NO Regulates Levels of Critical TCA Cycle Metabolites and Itaconate

Accumulation of TCA cycle metabolites citrate, succinate, and fumarate, as well as itaconate, is a hallmark of metabolic reprogramming of inflammatory macrophages (Arts et al., 2016; Infantino et al., 2011; Mills et al., 2018; Tannahill et al., 2013). Using untargeted metabolomics, we compared knockout models with controls and observed the predicted elevations in our stimulated NO-producing (Gch$^{fl/fl}$ and WT) macrophages (Figures 5A, 5B, 5C, S4, and S5). However, stimulated cells lacking NO (Gch$^{fl/fl}$Tie2cre and iNOS KO) even in the presence of BH_4 (iNOS KO) showed significantly elevated succinate (1.6-fold) but markedly less citrate, revealing an important effect of NO on inflammatory metabolites (Figures 5A, 5B, 5G, S4, and S5). Itaconate was also elevated 2.5-fold more in M(LPS+IFNγ) NO-deficient cells (Figure 5C). Itaconate is synthesized from citrate through the intermediate cis-aconitate by the enzyme cis-aconitate decarboxylase encoded by *Irg1* (Michelucci et al., 2013). We therefore examined whether increased levels of cis-aconitate decarboxylase could underlie the elevated itaconate but found no difference in protein levels in M(LPS+IFNγ) Gch$^{fl/fl}$ versus Gch$^{fl/fl}$Tie2cre cells by mass spectrometry (Figure 5D). This was confirmed by western blot analysis of cis-aconitate decarboxylase in both Gch$^{fl/fl}$Tie2cre and iNOS KO cells (Figure 5E) and occurred despite finding increased induction of *Irg1* transcript in stimulated Gch$^{fl/fl}$Tie2cre and iNOS KO cells (Figure 5G). Elevated itaconate levels were confirmed in M(LPS+IFNγ) Gch$^{fl.fl}$Tie2cre and iNOS KO cells compared with NO-replete

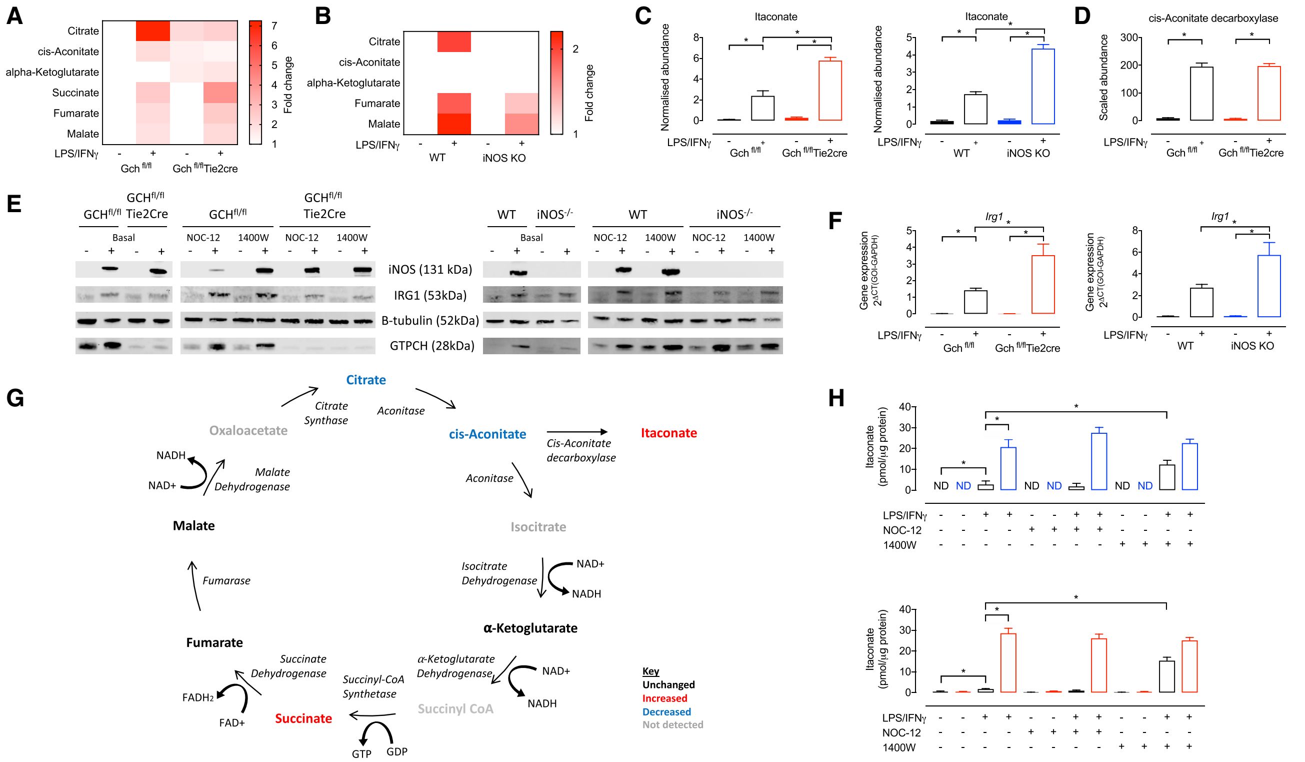

Figure 5. NO Modulates Levels of TCA Cycle Metabolites and Itaconate

(A and B) Fold change heatmaps of significantly changed ($p < 0.05$) metabolites relative to (A) Gch$^{fl/fl}$ or (B) WT unstimulated cells (n = 6, *itaconate fold change > 8) as measured using mass spectrometry.

(C) Itaconate metabolite abundance in Gch$^{fl/fl}$Tie2cre and iNOS KO cells measured using mass spectrometry (n = 6).

(D) Abundance of cis-aconitate decarboxylase protein in Gch$^{fl/fl}$Tie2cre cells measured using mass spectrometry (n = 4).

(E) Western blot analysis of cis-aconitate decarboxylase (IRG1), iNOS, and GTPCH protein (n = 4). β-tubulin was used as a loading control.

(F) *Irg1* gene expression in Gch$^{fl/fl}$Tie2cre and iNOS KO cells (n = 5).

(G) TCA cycle pathway showing changes (>20%) in abundance of enzymes and metabolites in LPS/IFNγ stimulated Gch$^{fl/fl}$Tie2cre versus Gch$^{fl/fl}$ macrophages.

(H) Itaconate measured using HPLC in Gch$^{fl.fl}$Tie2cre and iNOS KO cells treated with 1400W and NOC-12 (n = 4).

Data are mean + SEM; p values calculated using 2-way ANOVA with Tukey's post-test (*$p < 0.05$).

cells using high-performance liquid chromatography (HPLC) analysis (Figure 5H). In addition, HPLC analysis of M(LPS+IFNγ) Gch$^{fl/fl}$ and WT cells treated with 1400W showed significantly elevated itaconate, whereas NO donor treatment of all cells had no effect (Figure 5H).

Elevated citrate has previously been attributed to transcriptional repression of *Idh1* (Jha et al., 2015), and we therefore considered disruption of this "break point" as a potential cause of the depletion in citrate in the absence of NO. In accordance with previous reports (Jha et al., 2015; Tannahill et al., 2013), we did find repression of *Idh1* and *Idh2* gene expression in stimulated NO-synthesizing cells and also observed this in stimulated Gch$^{fl/fl}$Tie2cre and iNOS KO cells (Figures 6A and 6B). However, our proteomics data revealed that isocitrate dehydrogenase (IDH1) and IDH2 protein levels were unchanged by LPS/IFNγ stimulation (Figure 6C)—irrespective of BH_4 or NO production—and this was confirmed by western blotting for IDH1 (Figure 6D). IDH protein levels do not therefore explain changes in citrate in our model, nor underlie this "break point." In fact, we found no differences in the abundance of any TCA cycle enzymes in M(LPS+IFNγ) cells in the absence of BH_4 and NO (Figure S7), leading to the hypothesis that changes in their activity could account for the observed impact on metabolite levels. We therefore measured selected enzyme activities, which revealed significantly decreased IDH1 and IDH2 activity in stimulated WT but not Gch$^{fl/fl}$Tie2cre and iNOS KO cells compared with unstimulated Gch$^{fl/fl}$ or WT macrophages (Figure 6E). Interestingly, we also observed significantly decreased nitrosation at cysteine-133 in M(LPS+IFNγ) Gch$^{fl/fl}$Tie2cre macrophages compared with Gch$^{fl/fl}$ cells and significantly decreased nitrosation of cysteines-113, −154, and 418 in M(LPS+IFNγ) iNOS KO versus WT cells (Figure 6F). Furthermore, treatment of WT unstimulated macrophages with the IDH inhibitor GSK864 led to notable elevations in both citrate and itaconate (Figure 6G). Proteomics data showed a small but significant decrease in aconitase 1 protein in M(LPS+IFNγ) cells (Figure 6H), and dramatically decreased aconitase 1 and 2 activity was apparent in stimulated cells (Figure 6H), but these results were irrespective of BH_4 and NO production. Taken together, these results suggest that IDH but not aconitase activity is a key determinant of NO-dependent changes in TCA metabolites.

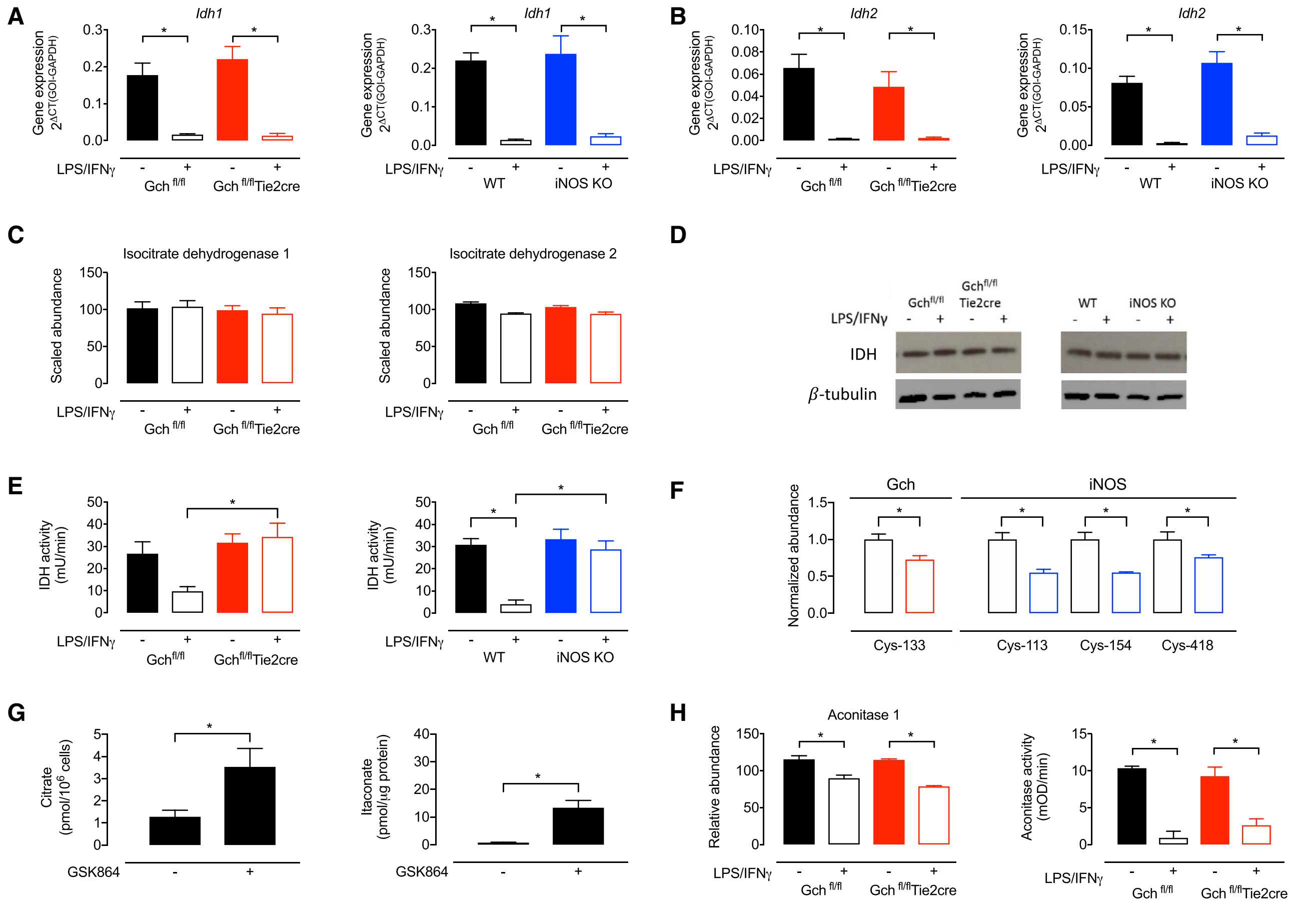

Figure 6. NO Modulates IDH Activity

(A) *Idh1* gene expression in $Gch^{fl/fl}$Tie2cre and iNOS KO cells (n = 5).

(B) *Idh2* gene expression in $Gch^{fl/fl}$Tie2cre and iNOS KO cells (n = 5).

(C) IDH1 and IDH2 protein abundance measured using mass spectrometry in $Gch^{fl/fl}$Tie2cre cells (n = 4).

(D) Western blot analysis of IDH1 protein in $Gch^{fl/fl}$Tie2cre and iNOS KO cells (n = 3).

(E) IDH1 and IDH2 combined activity in $Gch^{fl/fl}$Tie2cre and iNOS KO cells (n = 3).

(F) Normalized abundance of IDH peptides containing nitrosated cysteine residues, in samples from $Gch^{fl/fl}$Tie2cre and iNOS KO macrophages (n = 4).

(G) Citrate and itaconate levels in WT unstimulated macrophages treated with the IDH inhibitor GSK864 (n = 4).

(H) Abundance of aconitase 1 protein measured using mass spectrometry (n = 4) and aconitase 1 and 2 activity in $Gch^{fl/fl}$Tie2cre cells (n = 3–5).

Data are mean + SEM; p values calculated using 2-way ANOVA with Tukey's post-test (*p < 0.05).

NO-Deficient Peritoneal Macrophages Derived *In Vivo* Have Increased Itaconate and IL-1β

To see if the major results from macrophages differentiated from bone marrow precursor cells with colony-stimulating factors (CSFs) *in vitro* could be recapitulated from cells derived *in vivo*, we measured itaconate and IL-1β levels in thioglycolate-elicited peritoneal macrophages from both our murine models of NO deficiency. Fluorescence-activated cell sorting (FACS) analysis of freshly isolated peritoneal macrophages showed a high percentage of CD11+/F4/80+ cells, indicative of a relatively pure macrophage population (Figures 7A and 7B). We observed the expected inductions of iNOS protein in M(LPS+IFNγ) $Gch^{fl/fl}$, $Gch^{fl/fl}$Tie2cre, and WT cells but not iNOS KO cells, and elevated GTPCH in all M(LPS+IFNγ) cells apart from $Gch^{fl/fl}$Tie2cre (Figure 7C). NO_x was significantly decreased and barely detectable in $Gch^{fl/fl}$Tie2cre and iNOS KO macrophages, respectively (Figure 7D). In M(LPS+IFNγ) macrophages from both $Gch^{fl/fl}$Tie2cre and iNOS KO models, itaconate was increased ~2.5-fold compared to control (Figure 7E), despite unchanged amounts of IRG1 protein (Figure 7C). Similarly, levels of IL-1β were increased significantly in peritoneal macrophages deficient of NO (Figure 7F). While not exhaustive, these measures prove that the NO-dependent changes in immuno-metabolism and cytokine production in BMDMs are not simply artifacts of *in vitro* differentiation with CSFs.

BH_4- and NO-Deficient Macrophages Lead to Elevated Itaconate Accumulation in Two Distinct Models of Infection and Acute Inflammation *In Vivo*

Having demonstrated the NO-dependent modulation of the key anti-inflammatory metabolite itaconate in BMDMs, we aimed to recapitulate this key metabolic phenotype *in vivo*. First,

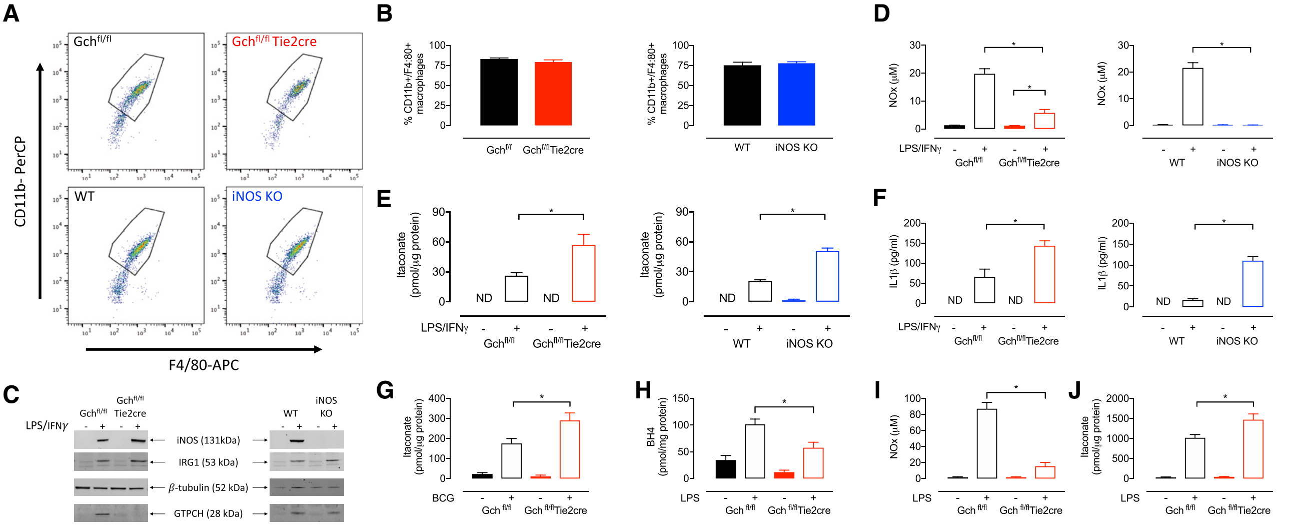

Figure 7. NO Modulates Levels of Itaconate in M(LPS+IFNγ) Peritoneal Macrophages, Mice Infected with BCG, and Mice Experiencing LPS-Induced Acute Endotoxaemia

(A) FACS analysis of peritoneal macrophages isolated from Gch$^{fl/fl}$Tie2cre and iNOS KO mice.

(B) The percentage of CD11b+/F4:80+ peritoneal macrophages isolated from Gch$^{fl/fl}$Tie2cre and iNOS KO mice measured using FACS (n = 4).

(C) Western blot analysis of iNOS, IRG1 and GTPCH protein in peritoneal macrophages stimulated with LPS/IFNγ (n = 4). β-tubulin was used as the loading control.

(D) NO_x measurements in medium supernatants from stimulated Gch$^{fl/fl}$Tie2cre and iNOS KO peritoneal macrophages (n = 4).

(E) Itaconate measured by HPLC in stimulated Gch$^{fl/fl}$Tie2cre and iNOS KO peritoneal macrophages (n = 4). (F) IL-1β secreted from stimulated Gch$^{fl/fl}$Tie2cre and iNOS KO peritoneal macrophages (n = 4).

(G) Itaconate measured by HPLC in lung tissue from Gch$^{fl/fl}$Tie2cre mice infected with BCG (n = 4–12).

(H–J) (H) BH_4 (H), NO_x (I), and itaconate (J) measured in lung tissue from Gch$^{fl/fl}$Tie2cre mice treated with 12.5 mg/kg LPS to induce acute endotoxaemia (n = 6). Data are mean + SEM; p values calculated using 2-way ANOVA with Tukey's post-test (*$p < 0.05$).

Gch$^{fl/fl}$Tie2cre mice were infected with *Mycobacterium bovis* Bacillus Calmette Guerin (BCG) and previously demonstrated to be deficient in BH_4 and NO_x (McNeill et al., 2018); we reveal that lungs from Gch$^{fl/fl}$Tie2cre mice also exhibit significantly elevated itaconate levels following BCG infection compared to WT (Figure 7G). Second, to more directly parallel our *in vitro* LPS activation, we then analyzed lung tissue taken from mice following LPS-induced endotoxaemia *in vivo* (Figure S8); lung tissue was analyzed and the levels of BH_4 and NO_x were confirmed to be decreased in tissues from LPS treated Gch$^{fl/fl}$Tie2cre versus Gch$^{fl/fl}$ mice (Figures 7H and 7I). Furthermore, in support of our findings in LPS-stimulated BMDMs and peritoneal macrophages *ex vivo*, LPS-induced endotoxaemia led to significantly elevated itaconate levels in lung tissue from NO-deficient Gch$^{fl/fl}$Tie2cre animals (Figure 7J). Together, these data demonstrate the translation of our *in vitro* analyses to confirm that BH_4 and NO levels modulate itaconate accumulation *in vivo*.

DISCUSSION

It is well established that BH_4 is required for generation of NO by iNOS in macrophages responding to inflammatory stimuli, and this is important for the destruction of pathogens, as part of the innate immune response. More recently, metabolic remodeling in macrophages has been recognized as a pivotal part of the pro-inflammatory switch, including the NO-mediated inhibition of mitochondrial oxidative phosphorylation. In the current study, we have shown that BH_4 modulates specific metabolic changes in inflammatory macrophages by the regulation of NO production. These include regulating respiratory function by changes in the abundance of critical N-module subunits in Complex I and changes in levels of the important TCA cycle metabolites (and inflammatory mediators) citrate, succinate, and itaconate. Taken together, we have demonstrated that inducible NO has a fundamental role in metabolic remodeling upon activation of macrophages.

Metabolic changes are intricately linked with the switch to a pro-inflammatory phenotype in macrophages, including the critical "Warburg"-like shift from oxidative metabolism to glycolytic ATP generation. NO is known to modulate ETC activity through nitrosation of Complex I and competitive inhibition of O_2-consuming Complex IV (Chouchani et al., 2013; Cleeter et al., 1994) and is responsible for inhibiting oxidative phosphorylation in both activated macrophages and dendritic cells (Everts et al., 2012; Van den Bossche et al., 2016). In accordance with these studies, we observed NO-dependent inhibition of Complex I and mitochondrial respiration. In addition, we have expanded the repertoire of NO-dependent mechanisms governing the ETC to include regulating the abundance of proteins making up the NADH-binding catalytic module (N-module) of Complex I. We propose that, upon stimulation, loss of essential N-module subunits renders Complex I incapable of binding and accepting electrons from NADH—thereby providing an insight

into the mechanisms that modulate the metabolic switch away from oxidative metabolism in activated macrophages. As *Ndufv2* mRNA was decreased in NO-synthesizing macrophages but maintained in iNOS KO cells, we hypothesize that NO regulates transcription of these subunits by an unknown mechanism. However, it is also possible that NO modulates the stability of these subunits, which are exposed in the mitochondrial matrix and might be sensitive to nitrosation.

Inhibition of mitochondrial respiration is not essential for macrophages to maintain their inflammatory polarization (Van den Bossche et al., 2016), in contrast with glycolysis that is upregulated and required for inflammatory cell survival and cytokine production (Krawczyk et al., 2010; Tannahill et al., 2013). In our NO-deficient M(LPS+IFNγ) macrophages, we found the expected elevation of glycolytic rates despite decreased abundance of several key proteins involved in glucose metabolism (GLUT-1, hexokinase-1 and −2, and PFKFB3) and the intracellular glycolytic metabolites fructose-1,6-bisphosphate, DAHP, and lactate. It was interesting to observe that extracellular lactate levels were also decreased, as this would usually correlate with glycolytic rates derived from extracellular acidification (Mookerjee et al., 2015). However, as glycolytic rate was determined at the point in time after overnight stimulation, whereas extracellular lactate measurements included accumulations overnight, our data might suggest that the time course for upregulating glycolysis is altered in NO-deficient M(LPS+IFNγ) cells.

In light of our results showing decreased levels of GLUT-1 and key glycolytic regulatory proteins (hexokinase-2 and PFKFB3), which are usually upregulated in NO-proficient inflammatory macrophages, it is important to consider alternate mechanisms upregulating glycolysis in NO-deficient cells. Although these protein changes appear to have inhibited the earlier part of glucose oxidation, causing decreased fructose-1,6-bisphosphate, increased abundance of triose phosphate isomerase, phosphoglycerate kinase, and 3-phosphoglycerate may indicate compensatory increased metabolism further along the pathway. In support of this, it is possible for metabolites from the pentose phosphate pathway and glycerol catabolism to feed in through dihydroxyacetone phosphate and glycerol-3-phosphate, and we have indications of increased glycerol metabolism through increased abundance of catabolic enzymes and decreased levels of glycerol-3-phosphate. Future studies will be interesting to dissect this, as well as the precise mechanisms behind these changes. In this report, we noted that several of the decreased proteins in our KO cells were known to be transcriptionally induced by HIF1α in WT inflammatory macrophages (Liu et al., 2016; Wang et al., 2017). Upon also observing decreased HIF1α in NO-deficient cells, we speculate that this may be one reason. NO has previously been shown to increase the stability and activity of HIF1α under non-hypoxic conditions (Kasuno et al., 2004; Sandau et al., 2001; Sumbayev et al., 2003), so this is a plausible hypothesis. However, as HIF1α levels are also modulated by molecules that include succinate, 4-octyl itaconate, and ROS, it is clear that we must consider that other such secondary changes are mechanistically responsible (Mills et al., 2016, 2018; Selak et al., 2005; Tannahill et al., 2013).

Accumulation of specific TCA cycle metabolites in activated macrophages has led to the discovery of their function in modulating inflammation (Arts et al., 2016; Infantino et al., 2011; Tannahill et al., 2013). Carbon flux analyses have identified two "breaks" in the cycle, at IDH and SDH (Jha et al., 2015), leading to elevations in citrate and succinate, respectively, and this was reproduced in our activated NO-synthesizing cells. A study identifying transcriptional repression of *Idh1* has suggested this as the reason for redirection of citrate into lipid and itaconate synthesis rather than isocitrate metabolism (Jha et al., 2015). Here, we also measured marked decreases in transcription of *Idh1* as well as *Idh2*, although our analysis revealed no subsequent changes in IDH protein levels. We also demonstrated that IDH and aconitase enzyme activities are dramatically decreased and therefore more likely to be responsible for the accumulation of citrate. Furthermore, we have shown that citrate levels are modulated by NO, as citrate was no longer elevated in macrophages incapable of producing NO. Although aconitase activity was decreased, IDH activity was maintained in NO-deficient M(LPS+IFNγ) cells and provides a possible avenue utilizing citrate in addition to increased itaconate production. This potentially explains the lack of citrate accumulation and is plausible, as NO has previously been shown to inhibit IDH activity through nitrosation (Lee et al., 2003; Yang et al., 2002), which was also shown to be decreased at several cysteine residues in NO-deficient M(LPS+IFNγ) cells.

Itaconate is one of the most highly induced metabolites following activation of BMDMs (Lampropoulou et al., 2016; Mills et al., 2018), and we have observed a 2.5-fold elevation in NO-deficient M(LPS+IFNγ) cells compared to NO-replete cells. In support of this finding, itaconate was shown to be elevated in WT cells treated with 1400W to decrease NO generation. Contrastingly, treatment of NO-deficient M(LPS+IFNγ) macrophages with a NO donor had no effect, possibly due to the inability of NOC-12 to enter the mitochondria and reach the intracellular target required to alter itaconate levels. Notably, we have also demonstrated the relevance of our *in vitro* findings by using *in vivo* models of BCG infection and LPS-induced endotoxaemia, where elevated itaconate levels were observed in lung tissues from leukocyte specific BH_4-deficient animals. Overall, these data suggest that NO can modulate inflammatory signaling by regulating itaconate levels, as itaconate is proven to be bactericidal but also an anti-inflammatory mediator (Bambouskova et al., 2018; Lampropoulou et al., 2016). Unchanged levels of the synthetic enzyme cis-aconitate decarboxylase indicated that increased itaconate could result from changes in enzyme activity, but as metabolites were only measured intracellularly, it may also be due to changes in itaconate release. It is also possible that the elevated protein response to IFNβ highlighted by our proteomics data is directing elevated itaconate production in NO-deficient cells, as Type I IFNs were shown to stimulate itaconate production in several studies (Mills et al., 2018; Naujoks et al., 2016; Tallam et al., 2016). Itaconate may also account for elevated succinate levels in our BH_4 and NO-deficient cells, as it is a proven inhibitor of SDH and leads to accumulation of succinate in stimulated macrophages (Cordes et al., 2016; Lampropoulou et al., 2016). This is another mechanism by which itaconate directs inflammatory signaling as succinate modulates

HIF1α-mediated IL-1β production and drives mitochondrial ROS generation (Mills et al., 2016; Tannahill et al., 2013). However, changes in succinate release are also a potential mechanism accounting for differences in intracellular levels.

The key pro-inflammatory mediators TNFα, IL-6, and IL-1β were all induced in activated NO-deficient as well as NO-synthesizing cells; however, IL-1β secretion was elevated alongside the abundance of proteins making up the inflammasome complex (NLRP3, AIM2, and caspase-1). The inflammasome cleaves pro-IL-1β into its mature secreted form, and NO is known to inhibit this complex through nitrosation (Hernandez-Cuellar et al., 2012; Mao et al., 2013; Mishra et al., 2013); thus the loss of NO signaling and decreased nitrosation of the inflammasome are likely to contribute to upregulated IL-1β secretion. In addition, the observed increased abundance of inflammasome proteins and transcription of *Il1b* must occur through NO-dependent and as yet unidentified mechanisms. HIF1α transcriptionally regulates *Il1b* in stimulated macrophages (Tannahill et al., 2013); however, as discussed, HIF1α is decreased in our models and so does not explain this finding. It is interesting to note that although *Il1b* gene expression was previously shown to be lower, it was still induced in HIF1α KO macrophages stimulated with LPS, thus suggesting that other mechanisms induce the expression of this gene and they warrant further study. More recently, NRF2 signaling was shown to repress IL-1β production in macrophages (Mills et al., 2018), and our group has previously demonstrated disrupted NRF2 signaling in stimulated macrophages devoid of NO due to BH_4 deficiency (Douglas et al., 2018; McNeill et al., 2015). NO-dependent changes in this pathway potentially determine increased *Il1b* transcription.

It is important to consider the potential translational relevance of our murine data in the human setting. It is well established that human macrophages do not produce such a fulminant NO response under many conditions of activation (Fang and Vazquez-Torres, 2002; Schneemann and Schoedon, 2002; Schneemann et al., 1993; Sharara et al., 1997; Vouldoukis et al., 1995). Part of this difference is in fact driven by their relatively low BH_4 content, due to exon skipping in the *PTPS* gene leading to a reduction in 6-pyruvoyl tetrahydropterin synthase (PTPS) in the BH_4 biosynthetic pathway (Leitner et al., 2003). Understanding how metabolic remodeling is altered in inflammatory macrophages in the context of low and high BH_4/NO therefore has profound implications for human macrophage biology and disease, and models such as the $Gch^{fl/fl}$Tie2cre knockout mice may in fact provide a better reflection of human biology.

In summary, growing evidence links metabolism to the production of inflammatory mediators (Mills et al., 2016, 2018; Tannahill et al., 2013), and in this study, we report that BH_4 modulates metabolic remodeling and immuno-metabolism through the modulation of NO signaling in murine macrophages responding to inflammatory stimuli, both *in vitro* and *in vivo*. It is important to consider the relevance of these findings in human macrophages, where NO generation appears to be more complex and BH_4 levels are lower (Fang and Vazquez-Torres, 2002; Leitner et al., 2003; Schneemann and Schoedon, 2002; Schneemann et al., 1993; Sharara et al., 1997; Vouldoukis et al., 1995), and this requires thorough investigation in the future. Nevertheless, we show that NO plays a unique and interesting role in regulating the balance of the key metabolites itaconate, succinate, and citrate, as well as the abundance of catalytic Complex I subunits in murine macrophages.

STAR★METHODS

Detailed methods are provided in the online version of this paper and include the following:

- KEY RESOURCES TABLE
- LEAD CONTACT AND MATERIALS AVAILABILITY
- EXERIMENTAL MODEL AND SUBJECT DETAILS
  - In Vivo Models
  - In Vitro Models
- METHOD DETAILS
  - Biopterin quantification by HPLC with electrochemical detection
  - Western blotting
  - NO_x measurements
  - Protein extraction and digestion
  - Tandem Mass Tag (TMT)-labeling of peptides
  - Mass spectrometric analysis of proteins
  - Metabolomics
  - mtDNA quantification
  - Quantitative real-time RT-PCR
  - Seahorse XF^{e}96 analysis of mitochondrial function
  - Seahorse XF^{e}96 analysis of glycolysis
  - Complex I activity assay
  - Lactate assay
  - Measurement of itaconate using HPLC
  - Isocitrate dehydrogenase activity assay
  - Aconitase activity assay
  - Measurement of citrate accumulation
  - Cytokine measurements by ELISA
  - Peritoneal macrophage recruitment
  - Organomercury enrichment and detection of S-nitrosated IDH by mass spectrometry
- QUANTIFICATION AND STATISTICAL ANALYSIS
  - Statistical analysis
  - Proteomic data analysis
  - Metabolomic data analysis
- DATA AND CODE AVAILABILITY

SUPPLEMENTAL INFORMATION

Supplemental Information can be found online at https://doi.org/10.1016/j.celrep.2019.06.018.

ACKNOWLEDGMENTS

This work was supported by the British Heart Foundation Intermediate Fellowship awarded to M.J.C. (FS/14/56/31049), British Heart Foundation Programme grants (RG/17/10/32859 and RG/12/5/29576), Wellcome Trust (090532/Z/09/Z), and the NIH Research (NIHR) Oxford Biomedical Research Centre. The authors would also like to acknowledge support from the BHF Centre of Research Excellence, Oxford (RE/13/1/30181 and RE/18/3/34214). The authors would like to thank the professors Harry Ischiropoulos and Paschalis-Thomas Doulias (University of Pennsylvania, USA) for the organomercury resin and advice on the organomercury enrichment of SNO proteins.

AUTHOR CONTRIBUTIONS

Concept: M.J.C. and K.M.C. Carried out experiments and analysis: J.B., M.D., T.N., E.M., A.S., S.C., A.H., A.S., E.S., S.D., and M.J.C. Resources and animal models: E.M., M.N., H.M., and K.M.C. Methods and analysis of proteomics: S.D., R.F., B.M.K., J.M., and M.J.C. Wrote the manuscript: J.B., A.S., and M.J.C. Funding: K.M.C. and M.J.C.

DECLARATION OF INTERESTS

The authors declare no competing interests.

Received: September 5, 2018
Revised: February 25, 2019
Accepted: June 5, 2019
Published: July 2, 2019

SUPPORTING CITATIONS

The following references appear in the Supplemental Information: Chen et al. (2001); Hu et al. (2003); Luo et al. (2006); Mathupala et al. (2001); Obach et al. (2004); Pelletier et al. (2012); Pescador et al. (2010); Riddle et al. (2000).

REFERENCES

Arts, R.J.W., Novakovic, B., Ter Horst, R., Carvalho, A., Bekkering, S., Lachmandas, E., Rodrigues, F., Silvestre, R., Cheng, S.-C., Wang, S.-Y., et al. (2016). Glutaminolysis and Fumarate Accumulation Integrate Immunometabolic and Epigenetic Programs in Trained Immunity. Cell Metab. *24*, 807–819.

Bailey, J., Shaw, A., Fischer, R., Ryan, B.J., Kessler, B.M., McCullagh, J., Wade-Martins, R., Channon, K.M., and Crabtree, M.J. (2017). A novel role for endothelial tetrahydrobiopterin in mitochondrial redox balance. Free Radic. Biol. Med. *104*, 214–225.

Bambouskova, M., Gorvel, L., Lampropoulou, V., Sergushichev, A., Loginicheva, E., Johnson, K., Korenfeld, D., Mathyer, M.E., Kim, H., Huang, L.H., et al. (2018). Electrophilic properties of itaconate and derivatives regulate the IκBζ-ATF3 inflammatory axis. Nature *556*, 501–504.

Chen, C., Pore, N., Behrooz, A., Ismail-Beigi, F., and Maity, A. (2001). Regulation of *glut1* mRNA by hypoxia-inducible factor-1. Interaction between H-ras and hypoxia. J. Biol. Chem. *276*, 9519–9525.

Chouchani, E.T., Methner, C., Nadtochiy, S.M., Logan, A., Pell, V.R., Ding, S., James, A.M., Cochemé, H.M., Reinhold, J., Lilley, K.S., et al. (2013). Cardioprotection by S-nitrosation of a cysteine switch on mitochondrial complex I. Nat. Med. *19*, 753–759.

Chuaiphichai, S., McNeill, E., Douglas, G., Crabtree, M.J., Bendall, J.K., Hale, A.B., Alp, N.J., and Channon, K.M. (2014). Cell-autonomous role of endothelial GTP cyclohydrolase 1 and tetrahydrobiopterin in blood pressure regulation. Hypertension *6*, 530–540.

Cleeter, M.W., Cooper, J.M., Darley-Usmar, V.M., Moncada, S., and Schapira, A.H. (1994). Reversible inhibition of cytochrome c oxidase, the terminal enzyme of the mitochondrial respiratory chain, by nitric oxide. Implications for neurodegenerative diseases. FEBS Lett. *345*, 50–54.

Clementi, E., Brown, G.C., Feelisch, M., and Moncada, S. (1998). Persistent inhibition of cell respiration by nitric oxide: crucial role of S-nitrosylation of mitochondrial complex I and protective action of glutathione. Proc. Natl. Acad. Sci. USA *95*, 7631–7636.

Cordes, T., Wallace, M., Michelucci, A., Divakaruni, A.S., Sapcariu, S.C., Sousa, C., Koseki, H., Cabrales, P., Murphy, A.N., Hiller, K., and Metallo, C.M. (2016). Immunoresponsive Gene 1 and Itaconate Inhibit Succinate Dehydrogenase to Modulate Intracellular Succinate Levels. J. Biol. Chem. *291*, 14274–14284.

Douglas, G., Hale, A.B., Patel, J., Chuaiphichai, S., Al Haj Zen, A., Rashbrook, V.S., Trelfa, L., Crabtree, M.J., McNeill, E., and Channon, K.M. (2018). Roles for endothelial cell and macrophage Gch1 and tetrahydrobiopterin in atherosclerosis progression. Cardiovasc. Res. *114*, 1385–1399.

Everts, B., Amiel, E., van der Windt, G.J.W., Freitas, T.C., Chott, R., Yarasheski, K.E., Pearce, E.L., and Pearce, E.J. (2012). Commitment to glycolysis sustains survival of NO-producing inflammatory dendritic cells. Blood *120*, 1422–1431.

Fang, F.C., and Vazquez-Torres, A. (2002). Nitric oxide production by human macrophages: there's NO doubt about it. Am. J. Physiol. Lung Cell. Mol. Physiol. *282*, L941–L943.

Guerrero-Castillo, S., Baertling, F., Kownatzki, D., Wessels, H.J., Arnold, S., Brandt, U., and Nijtmans, L. (2017). The Assembly Pathway of Mitochondrial Respiratory Chain Complex I. Cell Metab. *25*, 128–139.

Hernandez-Cuellar, E., Tsuchiya, K., Hara, H., Fang, R., Sakai, S., Kawamura, I., Akira, S., and Mitsuyama, M. (2012). Cutting edge: nitric oxide inhibits the NLRP3 inflammasome. J. Immunol. *189*, 5113–5117.

Hu, C.-J., Wang, L.-Y., Chodosh, L.A., Keith, B., and Simon, M.C. (2003). Differential roles of hypoxia-inducible factor 1alpha (HIF-1alpha) and HIF-2alpha in hypoxic gene regulation. Mol. Cell. Biol. *23*, 9361–9374.

Infantino, V., Convertini, P., Cucci, L., Panaro, M.A., Di Noia, M.A., Calvello, R., Palmieri, F., and Iacobazzi, V. (2011). The mitochondrial citrate carrier: a new player in inflammation. Biochem. J. *438*, 433–436.

Jha, A.K., Huang, S.C.-C., Sergushichev, A., Lampropoulou, V., Ivanova, Y., Loginicheva, E., Chmielewski, K., Stewart, K.M., Ashall, J., Everts, B., et al. (2015). Network integration of parallel metabolic and transcriptional data reveals metabolic modules that regulate macrophage polarization. Immunity *42*, 419–430.

Kasuno, K., Takabuchi, S., Fukuda, K., Kizaka-Kondoh, S., Yodoi, J., Adachi, T., Semenza, G.L., and Hirota, K. (2004). Nitric oxide induces hypoxia-inducible factor 1 activation that is dependent on MAPK and phosphatidylinositol 3-kinase signaling. J. Biol. Chem. *279*, 2550–2558.

Kelly, B., and O'Neill, L.A.J. (2015). Metabolic reprogramming in macrophages and dendritic cells in innate immunity. Cell Res. *25*, 771–784.

Krawczyk, C.M., Holowka, T., Sun, J., Blagih, J., Amiel, E., DeBerardinis, R.J., Cross, J.R., Jung, E., Thompson, C.B., Jones, R.G., and Pearce, E.J. (2010). Toll-like receptor-induced changes in glycolytic metabolism regulate dendritic cell activation. Blood *115*, 4742–4749.

Lampropoulou, V., Sergushichev, A., Bambouskova, M., Nair, S., Vincent, E.E., Loginicheva, E., Cervantes-Barragan, L., Ma, X., Huang, S.C.-C., Griss, T., et al. (2016). Itaconate Links Inhibition of Succinate Dehydrogenase with Macrophage Metabolic Remodeling and Regulation of Inflammation. Cell Metab. *24*, 158–166.

Lee, J.H., Yang, E.S., and Park, J.-W. (2003). Inactivation of $NADP^+$-dependent isocitrate dehydrogenase by peroxynitrite. Implications for cytotoxicity and alcohol-induced liver injury. J. Biol. Chem. *278*, 51360–51371.

Leitner, K.L., Meyer, M., Leimbacher, W., Peterbauer, A., Hofer, S., Heufler, C., Müller, A., Heller, R., Werner, E.R., Thöny, B., and Werner-Felmayer, G. (2003). Low tetrahydrobiopterin biosynthetic capacity of human monocytes is caused by exon skipping in 6-pyruvoyl tetrahydropterin synthase. Biochem. J. *373*, 681–688.

Liu, L., Lu, Y., Martinez, J., Bi, Y., Lian, G., Wang, T., Milasta, S., Wang, J., Yang, M., Liu, G., et al. (2016). Proinflammatory signal suppresses proliferation and shifts macrophage metabolism from Myc-dependent to HIF1α-dependent. Proc. Natl. Acad. Sci. USA *113*, 1564–1569.

Luo, F., Liu, X., Yan, N., Li, S., Cao, G., Cheng, Q., Xia, Q., and Wang, H. (2006). Hypoxia-inducible transcription factor-1α promotes hypoxia-induced A549 apoptosis via a mechanism that involves the glycolysis pathway. BMC Cancer *6*, 26.

Mao, K., Chen, S., Chen, M., Ma, Y., Wang, Y., Huang, B., He, Z., Zeng, Y., Hu, Y., Sun, S., et al. (2013). Nitric oxide suppresses NLRP3 inflammasome activation and protects against LPS-induced septic shock. Cell Res. *23*, 201–212.

Mathupala, S.P., Rempel, A., and Pedersen, P.L. (2001). Glucose catabolism in cancer cells: identification and characterization of a marked activation response of the type II hexokinase gene to hypoxic conditions. J. Biol. Chem. *276*, 43407–43412.

McNeill, E., Crabtree, M.J., Sahgal, N., Patel, J., Chuaiphichai, S., Iqbal, A.J., Hale, A.B., Greaves, D.R., and Channon, K.M. (2015). Regulation of iNOS function and cellular redox state by macrophage Gch1 reveals specific requirements for tetrahydrobiopterin in NRF2 activation. Free Radic. Biol. Med. *79*, 206–216.

McNeill, E., Stylianou, E., Crabtree, M.J., Harrington-Kandt, R., Kolb, A.-L., Diotallevi, M., Hale, A.B., Bettencourt, P., Tanner, R., O'Shea, M.K., et al. (2018). Regulation of mycobacterial infection by macrophage Gch1 and tetrahydrobiopterin. Nat. Commun. *9*, 5409.

Michelucci, A., Cordes, T., Ghelfi, J., Pailot, A., Reiling, N., Goldmann, O., Binz, T., Wegner, A., Tallam, A., Rausell, A., et al. (2013). Immune-responsive gene 1 protein links metabolism to immunity by catalyzing itaconic acid production. Proc. Natl. Acad. Sci. USA *110*, 7820–7825.

Mills, E.L., Kelly, B., Logan, A., Costa, A.S.H., Varma, M., Bryant, C.E., Tourlomousis, P., Däbritz, J.H.M., Gottlieb, E., Latorre, I., et al. (2016). Succinate Dehydrogenase Supports Metabolic Repurposing of Mitochondria to Drive Inflammatory Macrophages. Cell *167*, 457–470.e13.

Mills, E.L., Ryan, D.G., Prag, H.A., Dikovskaya, D., Menon, D., Zaslona, Z., Jedrychowski, M.P., Costa, A.S.H., Higgins, M., Hams, E., et al. (2018). Itaconate is an anti-inflammatory metabolite that activates Nrf2 via alkylation of KEAP1. Nature *556*, 113–117.

Mishra, B.B., Rathinam, V.A.K., Martens, G.W., Martinot, A.J., Kornfeld, H., Fitzgerald, K.A., and Sassetti, C.M. (2013). Nitric oxide controls the immunopathology of tuberculosis by inhibiting NLRP3 inflammasome-dependent processing of IL-1β. Nat. Immunol. *14*, 52–60.

Mookerjee, S.A., Goncalves, R.L.S., Gerencser, A.A., Nicholls, D.G., and Brand, M.D. (2015). The contributions of respiration and glycolysis to extracellular acid production. Biochim. Biophys. Acta. *1847*, 171–181.

Naujoks, J., Tabeling, C., Dill, B.D., Hoffmann, C., Brown, A.S., Kunze, M., Kempa, S., Peter, A., Mollenkopf, H.-J., Dorhoi, A., et al. (2016). IFNs Modify the Proteome of Legionella-Containing Vacuoles and Restrict Infection Via IRG1-Derived Itaconic Acid. PLoS Pathog. *12*, e1005408.

Obach, M., Navarro-Sabaté, A., Caro, J., Kong, X., Duran, J., Gómez, M., Perales, J.C., Ventura, F., Rosa, J.L., and Bartrons, R. (2004). 6-Phosphofructo-2-kinase (*pfkfb3*) gene promoter contains hypoxia-inducible factor-1 binding sites necessary for transactivation in response to hypoxia. J. Biol. Chem. *279*, 53562–53570.

Pelletier, J., Bellot, G., Gounon, P., Lacas-Gervais, S., Pouysségur, J., and Mazure, N.M. (2012). Glycogen Synthesis is Induced in Hypoxia by the Hypoxia-Inducible Factor and Promotes Cancer Cell Survival. Front. Oncol. *2*, 18.

Pescador, N., Villar, D., Cifuentes, D., Garcia-Rocha, M., Ortiz-Barahona, A., Vazquez, S., Ordoñez, A., Cuevas, Y., Saez-Morales, D., Garcia-Bermejo, M.L., et al. (2010). Hypoxia promotes glycogen accumulation through hypoxia inducible factor (HIF)-mediated induction of glycogen synthase 1. PLoS One *5*, e9644.

Riddle, S.R., Ahmad, A., Ahmad, S., Deeb, S.S., Malkki, M., Schneider, B.K., Allen, C.B., and White, C.W. (2000). Hypoxia induces hexokinase II gene expression in human lung cell line A549. Am. J. Physiol. Lung Cell. Mol. Physiol. *278*, L407–L416.

Sandau, K.B., Fandrey, J., and Brüne, B. (2001). Accumulation of HIF-1alpha under the influence of nitric oxide. Blood *97*, 1009–1015.

Schneemann, M., and Schoedon, G. (2002). Species differences in macrophage NO production are important. Nat. Immunol. *3*, 102.

Schneemann, M., Schoedon, G., Hofer, S., Blau, N., Guerrero, L., and Schaffner, A. (1993). Nitric oxide synthase is not a constituent of the antimicrobial armature of human mononuclear phagocytes. J. Infect. Dis. *167*, 1358–1363.

Selak, M.A., Armour, S.M., MacKenzie, E.D., Boulahbel, H., Watson, D.G., Mansfield, K.D., Pan, Y., Simon, M.C., Thompson, C.B., and Gottlieb, E. (2005). Succinate links TCA cycle dysfunction to oncogenesis by inhibiting HIF-α prolyl hydroxylase. Cancer Cell *7*, 77–85.

Sharara, A.I., Perkins, D.J., Misukonis, M.A., Chan, S.U., Dominitz, J.A., and Weinberg, J.B. (1997). Interferon (IFN)-alpha activation of human blood mononuclear cells in vitro and in vivo for nitric oxide synthase (NOS) type 2 mRNA and protein expression: possible relationship of induced NOS2 to the anti-hepatitis C effects of IFN-alpha in vivo. J. Exp. Med. *186*, 1495–1502.

Sumbayev, V.V., Budde, A., Zhou, J., and Brüne, B. (2003). HIF-1 alpha protein as a target for S-nitrosation. FEBS Lett. *535*, 106–112.

Tallam, A., Perumal, T.M., Antony, P.M., Jäger, C., Fritz, J.V., Vallar, L., Balling, R., Del Sol, A., and Michelucci, A. (2016). Gene Regulatory Network Inference of Immunoresponsive Gene 1 (IRG1) Identifies Interferon Regulatory Factor 1 (IRF1) as Its Transcriptional Regulator in Mammalian Macrophages. PLoS One *11*, e0149050.

Tannahill, G.M., Curtis, A.M., Adamik, J., Palsson-McDermott, E.M., McGettrick, A.F., Goel, G., Frezza, C., Bernard, N.J., Kelly, B., Foley, N.H., et al. (2013). Succinate is an inflammatory signal that induces IL-1β through HIF-1α. Nature *496*, 238–242.

Tayeh, M.A., and Marletta, M.A. (1989). Macrophage oxidation of L-arginine to nitric oxide, nitrite, and nitrate. Tetrahydrobiopterin is required as a cofactor. J. Biol. Chem. *264*, 19654–19658.

Van den Bossche, J., Baardman, J., Otto, N.A., van der Velden, S., Neele, A.E., van den Berg, S.M., Luque-Martin, R., Chen, H.-J., Boshuizen, M.C.S., Ahmed, M., et al. (2016). Mitochondrial Dysfunction Prevents Repolarization of Inflammatory Macrophages. Cell Rep. *17*, 684–696.

Vouldoukis, I., Riveros-Moreno, V., Dugas, B., Ouaaz, F., Bécherel, P., Debré, P., Moncada, S., and Mossalayi, M.D. (1995). The killing of Leishmania major by human macrophages is mediated by nitric oxide induced after ligation of the Fc epsilon RII/CD23 surface antigen. Proc. Natl. Acad. Sci. USA *92*, 7804–7808.

Wang, T., Liu, H., Lian, G., Zhang, S.-Y., Wang, X., and Jiang, C. (2017). HIF1α-Induced Glycolysis Metabolism Is Essential to the Activation of Inflammatory Macrophages. Mediators Inflamm. *2017*, 9029327.

Yang, E.S., Richter, C., Chun, J.-S., Huh, T.-L., Kang, S.-S., and Park, J.-W. (2002). Inactivation of NADP(+)-dependent isocitrate dehydrogenase by nitric oxide. Free Radic. Biol. Med. *33*, 927–937.

STAR★METHODS

KEY RESOURCES TABLE

REAGENT or RESOURCE	SOURCE	IDENTIFIER
Antibodies		
Anti-iNOS (1:1000)	Abcam	Cat# ab49999, RRID:AB_881438
Anti-GTPCH (1:5000)	Prof. S. Gross, Cornell	N/A
Anti-β-tubulin (1:20,000)	Abcam	Cat# 6046, RRID:AB_2210370
Anti-Ndufv2 (1:1000)	Abcam	Cat# ab183715, RRID:AB_2687934
Anti-HIF1α (1:500)	Novus	Cat# NB100-449, RRID:AB_10001045
Anti-hexokinase-2 (1:1000)	Cell signaling	Cat# 2867S, RRID:AB_2232946
Anti-IDH1 (1:1000)	Cell signaling	Cat# 8137S, RRID:AB_10950504
DuoSet ELISA IL-1β	R&D systems	Cat# DY401-05
DuoSet ELISA TNFα	R&D systems	Cat# DY410-05
DuoSet ELISA IL-6	R&D systems	Cat# DY406-05
DuoSet ELISA IL-10	R&D systems	Cat# DY417-05
Cis-aconitate decarboxylase (IRG1) antibody	Eurogentec	N/A
Chemicals, Peptides, and Recombinant Proteins		
Interferon γ	PeproTech	Cat# 315-05
Lipopolysaccharide	Sigma	Cat# L4391
Macrophage colony stimulating factor	Peprotech	Cat# 315-02
Granulocyte macrophage colony stimulating factor	Peprotech	Cat# 315-03
GSK864	Sigma	SML1757
Critical Commercial Assays		
Isocitrate dehydrogenase activity assay	Abcam	Cat# ab102528
Aconitase activity assay	Abcam	Cat# ab109712
Citrate assay kit	Sigma	MAK057
Deposited Data		
Gchfl/flTie2cre Raw and normalized TMT data	PRIDE	PRIDE: PXD010628
Experimental Models: Organisms/Strains		
Mouse: Nos2−/− (Nos2^{tm1Lau})	The Jackson Laboratory	Cat#002609
Mouse: Gch1$^{fl/fl}$Tie2cre		N/A
Oligonucleotides		
Taqman murine Il1b assay	Applied biosystems	Mm00434228_m1
Taqman murine Ndufv2 assay	Applied biosystems	Mm01239727_m1
Taqman murine Slc2a1 assay	Applied biosystems	Mm00441480_m1
Taqman murine Irg1 assay	Applied biosystems	Mm01224532_m1
Taqman murine Idh1 assay	Applied biosystems	Mm00516030_m1
Taqman murine Idh2 assay	Applied biosystems	Mm00612429_m1
Software and Algorithms		
Cytoscape v3.6.0		https://cytoscape.org/
Graph Pad Prism 7	Graph Pad	https://www.graphpad.com/scientific-software/prism/
Other		
Phenylmercury resin	Gift from Professor Harry Ischiropoulos, UPENN, USA	N/A

LEAD CONTACT AND MATERIALS AVAILABILITY

Further information and requests for resources and reagents should be directed to and will be fulfilled by the Lead Contact, Dr Mark J. Crabtree (mark.crabtree@well.ox.ac.uk).

EXERIMENTAL MODEL AND SUBJECT DETAILS

In Vivo Models

Animal details

All animal procedures were approved and carried out in accordance with the University of Oxford ethical committee and the UK Home Office Animals (Scientific Procedures) Act 1986. All procedures conformed with the Directive 2010/63/EU of the European Parliament.

We have generated a *Gch1* conditional knockout (floxed) allele using Cre/loxP strategy as described previously (Chuaiphichai et al., 2014; McNeill et al., 2015). *Gch1*$^{fl/fl}$ animals were bred with Tie2cre transgenic mice to produce *Gch1*$^{fl/fl}$Tie2cre mice where *Gch1* is deleted in endothelial cells and bone marrow-derived cells. The Tie2cre transgene is active in the female germline. Consequently, only male animals are used to establish breeding pairs to maintain conditional expression. Experiments were performed using bone marrow isolated from 10-16 weeks old adult male and female *Gch1*$^{fl/fl}$Tie2cre (referred to as Gch$^{fl/fl}$Tie2cre) and their Gch1$^{fl/fl}$ (Gch$^{fl/fl}$) littermates on a pure (> 10 generations) C57BL6/J background. Mice were genotyped according to the published protocol (Chuaiphichai et al., 2014; McNeill et al., 2015). Nos2$^{-/-}$ (Nos2^{tm1Lau}) (iNOS KO) and wild-type C57BL6/J mice were purchased from The Jackson Laboratory.

BCG intranasal infection

For intranasal (i.n.) infection, adult female mice (> 10-week old) were sedated using IsoFlo (Oxford University Veterinary Services; UK) and inoculated with 5×10^6 CFU BCG through the nostrils (25 μl/nostril). Four weeks after infection, mice were sacrificed and lungs were aseptically removed. Organs were homogenized in re-inforced tubes with ceramic beads containing 1 ml PBS using Precellys 24 (Stretton Scientific, UK). These samples were gathered as part of a previous study, and samples were reused for metabolic analysis to study the role of nitric oxide in metabolite accumulation accordingly (McNeill et al., 2015).

In vivo *endotoxemia*

Endotoxemia was induced under brief anesthesia (2% isoflurane; by air pump). Endotoxemia was induced in adult female mice (> 10-week old) by intravenous injection into the tail vein of lipopolysaccharide (LPS; 12.5 mg/kg) from *Salmonella* typhimurium (Sigma, L7261) as described previously. Mice were housed with access to a heating pad after injection. Prior to harvest, 24hrs after injection, mice were anesthetized under isoflurane (2% by air pump), and core temperature was recorded by a rectal probe. A venous blood sample was drawn from the inferior vena cava under terminal anesthesia for blood biochemistry. Blood biochemistry was assessed immediately from 100 μL venous blood using a hand-held iSTAT point-of-care analyzer (Abbott Laboratories), with EC8+ cartridges (Abbott Laboratories).

In Vitro Models

Culturing primary bone marrow derived macrophages

Bone marrow was obtained by flushing the femur and tibia of adult mice with PBS. A single cell suspension was prepared by passing the bone marrow through a 70 μm cell strainer. Cells were then cultured on non-tissue culture treated plastic at 750,000 cells/well of 6 well plate or 3x10^6 cell/10 cm dishes for 7 days in DMEM:F12 (ThermoFisher Scientific) supplemented with penicillin (100 U/ml) and streptomycin (100 ng/ml, Sigma), Ultra-low endotoxin fetal bovine serum (5%, Biowest), l-glutamine (5 mmol/liter, Sigma), and recombinant macrophage colony-stimulating factor (MCSF) protein (25ng/ml, Peprotech) at 37°C and 5% CO2. On day 6 recombinant granulocyte-MCSF (GMCSF) protein (50ng/ml, Peprotech) was added to the cells.

Stimulation of bone marrow-derived macrophages

Following differentiation cells were washed once with warm PBS and the media replaced with DMEM:F12 (ThermoFisher Scientific) supplemented with penicillin (100 U/ml) and streptomycin (100 ng/ml, Sigma), Ultra-low endotoxin fetal bovine serum (2%, Biowest), l-glutamine (5 mmol/liter, Sigma), MCSF (25ng/ml, Peprotech) and GMCSF (50ng/ml) before stimulation with IFNγ (10 ng/ml, Peprotech) and LPS (100 ng/ml, Sigma) for 16 hours, with parallel wells left unstimulated. Exceptionally, for extracellular flux assays cells were re-plated onto tissue culture treated Seahorse XF^{e}96 well plates 2 hours before stimulation. After 16 hours cell pellets, and cell culture supernatants were collected, or the cells subjected to biochemical analysis.

For cells treated with NOC-12 (EMD Millipore, 487955) and 1400W dihydrochloride (Sigma, W4262), either 500 μM of NOC-12 or 10 μM of 1400W were added to media simultaneous to LPS/IFNγ stimulation. Both NOC-12 and 1400W were solubilised with sterile PBS to a stock concentration of 56.8mM and 40mM respectively.

METHOD DETAILS

Biopterin quantification by HPLC with electrochemical detection

BH_4, BH_2, and biopterin levels in cell and mitochondrial lysates were determined by HPLC followed by electrochemical and fluorescent detection, as described previously. Macrophage pellets were resuspended in PBS (50 mmol/liter), pH 7.4, containing

dithioerythritol (1 mmol/liter) and EDTA (100 μmol/liter) and subjected to three freeze-thaw cycles. Following centrifugation (15 min at 17,000 g, 4°C), the samples were transferred to new, cooled microtubes and precipitated with ice-cold extraction buffer containing phosphoric acid (1 mol/liter), trichloroacetic acid (2 mol/liter), and dithioerythritol (1 mmol/liter). The samples were vigorously mixed and then centrifuged for 15 minutes at 17,000 g, 4°C. The samples were injected onto an isocratic HPLC system and quantified using sequential electrochemical (Coulochem III, ESA Inc.) and fluorescence (Jasco) detection. HPLC separation was performed using a 250 mm, ACE C-18 column (Hichrom) and mobile phase comprising of sodium acetate (50 mmol/liter), citric acid (5 mmol/liter), EDTA (48 μmol/liter), and dithioerythritol (160 μmol/liter) (pH 5.2) (all ultrapure electrochemical HPLC grade), at a flow rate of 1.3 ml/min. Background currents of +500 μA and −50 μA were used for the detection of BH_4 on electrochemical cells E1 and E2, respectively. 7,8-BH_2 and biopterin were measured using a Jasco FP2020 fluorescence detector. Quantification of BH_4, BH_2, and biopterin was made by comparison with authentic external standards and normalized to sample protein content.

Western blotting

Cell lysates were prepared by homogenization in ice-cold CelLytic M buffer (Sigma) containing protease inhibitor cocktail (Roche Applied Science). Lysates were centrifuged at 17,000 g for 10 minutes at 4°C, and samples were prepared using LDS sample buffer (Invitrogen). Western blotting was carried out using standard techniques with antibodies outlined in the Key Resources Table. Exceptionally the IRG1 antibody (working concentration is 1:1000) is a polyclonal rabbit antibody custom made by Eurogentec Ltd (Southampton) following rabbit immunisation and affinity purification.

NO_x measurements

The levels of nitrite/nitrate (NOx) produced by bone marrow derived macrophages from $Gch^{fl/fl}$Tie2cre and $Gch^{fl/fl}$ control mice were determined using the CLD88 NO analyzer (Ecophysics), as previously described (McNeill et al., 2015). Briefly, media was collected from activated and control BMDMs and stored at −80°C for analysis. All samples were then thawed on ice, and NO liberation from 100 ul sample media (following chemical reduction by vanadium(III) chloride (VCl3) dissolved in HCl) was measured via a chemiluminescent reaction with ozone. Quantification of NOx accumulation was obtained by comparison with external standards and normalized to protein concentration, determined by the bicinchoninic acid (BCA) protein assay.

Protein extraction and digestion

Cell pellets were lysed in RIPA buffer by freeze thawing on dry ice, and centrifuged at 17,000 g for 10 minutes at 4°C. Extraction, digestion and labeling of proteins were carried out using solutions prepared in triethylammonium bicarbonate buffer (TEAB, 1 mM). Dithiothreitol (DTT, 5 mM) was added to supernatants and incubated for 30 minutes at room temperature to reduce samples. Iodoacetamide (20 mM) was added and incubated for 30 minutes at room temperature to alkylate samples before protein was precipitated out using a methanol/chloroform extraction method. In brief, relative to sample volumes, 3 volumes of methanol and 0.5 volumes of chloroform were added to samples and vortexed, before adding a further 2.25 volumes of water and vortexing again. Samples were centrifuged at 17,000 g for 1 minute and the upper aqueous phase was removed. More methanol was added, and samples were vortexed and centrifuged at 17,000 g for 2 minutes. Supernatants were discarded and the resulting protein pellet was resuspended in urea (6 mol/liter) and subsequently diluted to 1 mol/liter urea with water. Sequencing grade modified trypsin (Promega) was added at a ratio of 1:50 relative to protein content, and allowed to digest samples over night at 37°C. The following day formic acid (< 1%) was added to stop trypsinolysis, samples were desalted using C18 Sep-Pack cartridges (Waters), vacuum dried (Speedvac, ThermoFisher Scientific) and stored at −80°C.

Tandem Mass Tag (TMT)-labeling of peptides

Digested proteins were resuspended in TEAB (50 mmol/liter). Peptide concentrations were determined using a Pierce™ Quantitative Colorimetric Peptide Assay kit (ThermoFisher Scientific) and calculated against a standard curve of known peptide concentrations with a plate reader set at an absorbance of 490 nm. 100 ug of each sample was taken, made up to 100 ug/ul in TEAB (50 mM) and labeled using TMT10plex™ Isobaric Label Reagent Sets (ThermoFisher Scientific) following the manufacturers protocol. TMT labels were resuspended in acetonitrile, added to samples and incubated at room temperature for 1 hour. Hydroxylamine (5%) was added for 15 minutes to quench the labeling reactions. The experimental design required 16 samples (4 each of untreated $Gch^{fl/fl}$, untreated $Gch^{fl/fl}$Tie2cre, $M^{LPS/IFN\gamma}$ $Gch^{fl/fl}$ and $M^{LPS/IFN\gamma}$ $Gch^{fl/fl}$Tie2cre) which were therefore split over 2x TMT10plex™ kits (8 samples in each), with 2 labels used to link the sample sets and formed of identical pools containing equal quantities of all samples. Samples labeled using Kit 1 and Kit 2 were pooled separately and desalted using C18 Sep-Pack cartridges (Waters), vacuum dried and stored at −80°C.

Mass spectrometric analysis of proteins

TMT-labeled peptides resuspended in 2% acetonitrile 0.1% trifluoroacetic acid were fractionated through an XBridge BEH C18 2.5um XP column (Waters) using HPLC and a high pH fractionation protocol. Mobile phases consisted of water (pH 10, Buffer A) and acetonitrile (90%, pH 10, Buffer B) and ran at 1% Buffer B for 12 mins, followed by a linear gradient to 35% Buffer B over 72 minutes and finally 95% Buffer B for 10 minutes. Eluted peptides were collected every 2 minutes and concatenated at the end to form 8 fractions which were vacuum dried and then resuspended in acetonitrile (2%) and TFA (0.1%). Fractions were analyzed by LC-MS/MS using a

Dionex Ultimate 3000 nanoUPLC coupled to an Orbitrap Fusion Lumos mass spectrometer (Thermo Scientific). Peptides were separated on a 50 cm EASY-Spray C18 column (75 μm x 500 mm, 2 μm particle size; Thermo Scientific) over a 120 minute gradient from 2% to 35% acetonitrile in 0.1% formic acid, 5% DMSO. The mass spectrometer was set to perform data-dependant acquisition in SPS-MS^3 mode with a cycle time of 4 s. Detailed mass spectrometer settings are listed in the table below.

**MS1**	
Detector	Orbitrap, 120k
Scan range	380 - 1500 m/z
AGC target	200000
Injection time	50 ms
**MS2**	
Detector	IonTrap, Rapid
First mass	120
AGC target	10000
Injection time	50 ms
Isolation width	0.7 m/z
Fragmentation	CID 35%
**MS3**	
Detector	Orbitrap, 60k
Scan range	120 - 500 m/z
AGC target	100000
Injection time	120 ms
SPS notches	3
Fragmentation	HCD 65%

Metabolomics

Cell were harvested and pellets immediately frozen on dry ice and stored at −80°C prior to shipping to Metabolon on dry ice. Metabolon measured metabolites using mass spectrometry, as outlined below.

Sample Preparation

Samples were prepared using the automated MicroLab STAR® system from Hamilton Company. Several recovery standards were added prior to the first step in the extraction process for QC purposes. To remove protein, dissociate small molecules bound to protein or trapped in the precipitated protein matrix, and to recover chemically diverse metabolites, proteins were precipitated with methanol under vigorous shaking for 2 min (Glen Mills GenoGrinder 2000) followed by centrifugation. The resulting extract was divided into five fractions: two for analysis by two separate reverse phase (RP)/UPLC-MS/MS methods with positive ion mode electrospray ionization (ESI), one for analysis by RP/UPLC-MS/MS with negative ion mode ESI, one for analysis by HILIC/UPLC-MS/MS with negative ion mode ESI, and one sample was reserved for backup. Samples were placed briefly on a TurboVap® (Zymark) to remove the organic solvent. The sample extracts were stored overnight under nitrogen before preparation for analysis.

Ultrahigh Performance Liquid Chromatography-Tandem Mass Spectroscopy (UPLC-MS/MS):

All methods utilized a Waters ACQUITY ultra-performance liquid chromatography (UPLC) and a Thermo Scientific Q-Exactive high resolution/accurate mass spectrometer interfaced with a heated electrospray ionization (HESI-II) source and Orbitrap mass analyzer operated at 35,000 mass resolution. The sample extract was dried then reconstituted in solvents compatible to each of the four methods. Each reconstitution solvent contained a series of standards at fixed concentrations to ensure injection and chromatographic consistency. One aliquot was analyzed using acidic positive ion conditions, chromatographically optimized for more hydrophilic compounds. In this method, the extract was gradient eluted from a C18 column (Waters UPLC BEH C18-2.1x100 mm, 1.7 μm) using water and methanol, containing 0.05% perfluoropentanoic acid (PFPA) and 0.1% formic acid (FA). Another aliquot was also analyzed using acidic positive ion conditions, however it was chromatographically optimized for more hydrophobic compounds. In this method, the extract was gradient eluted from the same afore mentioned C18 column using methanol, acetonitrile, water, 0.05% PFPA and 0.01% FA and was operated at an overall higher organic content. Another aliquot was analyzed using basic negative ion optimized conditions using a separate dedicated C18 column. The basic extracts were gradient eluted from the column using methanol and water, however with 6.5mM Ammonium Bicarbonate at pH 8. The fourth aliquot was analyzed via negative ionization following elution from a HILIC column (Waters UPLC BEH Amide 2.1x150 mm, 1.7 μm) using a gradient consisting of water and acetonitrile with 10mM Ammonium Formate, pH 10.8. The MS analysis alternated between MS and data-dependent MS^n scans using dynamic exclusion. The scan range varied slighted between methods but covered 70-1000 m/z. Raw data files are archived and extracted as described below.

mtDNA quantification

DNA was extracted from macrophage cell pellets using QIAGEN blood and tissue kit according to manufacturer's instructions. DNA concentrations were measured by nanodrop and adjusted to 10ng/μl with nuclease free water. Quantification of mitochondrial DNA copy number was performed by quantitative PCR of two nuclear encoded genes (*Actb* and *Sdha*), and two mitochondrially encoded genes (*Cytb* and *mtCo2*). Quantitative PCR reactions were prepared with 20ng of DNA, 1x iTAQ Universal SYBR Green Supermix (Biorad), and 0.06μl of the relevant forward and reverse primers at 50μM concentration (ActbF – CTGCCTGACG GCCAGG, ActR – GAAAAGAGCCTCAGGGCA, SdhaF – TACTACAGCCCCAAGTCT, SdhaR – TGGACCCATCTTCTATGC, CytbF – CCACTTCATCTTACCATTTATTATCGC, CytbR – TTTTATCTGCATCTGAGTTTAATCCTGT, mtCo2F – CTACAAGACGC CACAT, mtCo2R – GAGAGGGGAGAGCAAT) (Bailey et al., 2017). Final volume was adjusted to 10μl with nuclease free water. PCR reaction was performed on Biorad CFX96 and was initiated at 95°C for 4 minutes followed by 40 cycles of 95°C for 5 s and 60°C for 30 s. Mitochondrial DNA copy number was calculated by averaging the ΔCt values of each pair of genes (*Actb* versus *Cytb* and *Sdha* versus *mtCo2*) in the calculation: mtDNA copy number = $2*((2\hat{}(Ct_{Actb}-Ct_{Cytb}) + 2\hat{}(Ct_{Sdha}-Ct_{mtCo2}))/2)$.

Quantitative real-time RT-PCR

Total RNA was isolated using RNeasy kits (QIAGEN) and reverse transcribed to cDNA using QuantiTect Reverse Transcriptase (QIAGEN). Quantitative real-time PCR was performed with 25 ng of cDNA on an iCycler IQ real time detection system (Bio-Rad Laboratories Ltd., UK). Gene expression was determined using TaqMan Gene Expression Assays (Applied Biosystems, UK) relative to the level of the house keeping gene GAPDH using real time RT-PCR.

Seahorse XF^{e}96 analysis of mitochondrial function

On day 7 of culture macrophages were plated at a density of 8×10^4 cells per well into XF^{e}96 microplates. Cells were left to attach for 2 hours before stimulation with LPS/IFNγ if required, for 16 hours. Extracellular flux analysis was then performed. One hour prior to the assay, cells were washed and the culture medium was replaced with Seahorse XF Base Medium (modified DMEM with Phenol Red, pH 7.4; Agilent), supplemented with glucose (10 mM, Sigma), glutamine (2 mM, Sigma), and sodium pyruvate (2 mM, Sigma), before being incubated at 37°C, at atmospheric CO_2 levels. Oxygen consumption rate (OCR) and extracellular acidification rate (ECAR) were measured using the XF^{e}96 analyzer (Agilent). 3 base-line OCR measurements were taken followed by 3 measurements after sequential injection of the following compounds from the XF Cell Mito Stress Test Kit (Agilent): oligomycin (2 μM, injection port A), FCCP (2 μM, injection port B), and combined antimycin A (0.5 μM), and rotenone (0.5 μM) (injection port C). Cells were measured in 10 replicate wells and all Seahorse was data normalized to cell number. For calculations (detailed in the table below) the third reading in each case was used as the most stable point, with the exception of the highest reading following addition of FCCP.

Respiratory function parameter	Calculation
Basal OCR	Base-line – Rotenone/antimycin A
ATP-linked OCR	Base-line - Oligomycin
Maximal OCR	FCCP - Rotenone/antimycin A

Seahorse XF^{e}96 analysis of glycolysis

Glycolytic activity was determined using the simultaneous ECAR readings generated during the above XF Cell Mito Stress Test, and included the subsequent addition of 2-deoxyglucose (100 mM, Sigma) injected through port D. The third ECAR measurement following the 2-deoxyglucose injection was subtracted from the third base-line ECAR reading to calculate 'basal glycolysis'.

Second, the Seahorse XF Glycolytic Rate Assay protocol was used to generate a more robust measurement of glycolysis. Cells were plated and stimulated as above for the XF Cell Mito Stress Test. One hour prior to the assay, cells were washed and the culture medium was replaced with Seahorse XF Base Medium without Phenol Red (modified DMEM, pH 7.4; Agilent), supplemented with glucose (10 mM, Sigma), glutamine (2 mM, Sigma), sodium pyruvate (1 mM, Sigma), and HEPES (5 mM, Agilent) before being incubated at 37°C, at atmospheric CO_2 levels. Immediately prior to the assay this media was again replaced. 3 base-line OCR and ECAR measurements were taken followed by 3 measurements after injection of combined antimycin A (0.5 μM), and rotenone (0.5 μM) (injection port A, Mito Stress Test Kit), and 5 measurements after injection of 2-deoxyglucose (50 mM, Sigma). ECAR measurements were converted into proton efflux rates (PER) and mitochondrial OCR (base-line OCR – rotenone/antimycin A) was used to determine the PER attributed to glycolysis (glycoPER) and mitochondrial (mitoPER) acidification using the Agilent Seahorse XF Glycolytic Rate Assay Report Generator and the Buffer (BF, 2.2) and CO_2 contribution factors (CCF, 0.61) predetermined by Agilent.

Complex I activity assay

Complex I activity was measured in mitochondria isolated using Qproteome mitochondrial isolation kit (QIAGEN). 25 μg of mitochondrial protein was resuspended in 1x native sample buffer (Invitrogen), 2% digitonin (supercomplexes) or 2% DDM (n-dodecyl-β-D-maltoside) (isolated complex I), and 1x protease inhibitors (Roche) followed by 1 hour incubation on ice before final addition of 0.5% G-250 sample additive. Prepared samples were run on 3%–12% bis-tris native gels under native conditions at 150 V for 30 minutes in

1x native cathode buffer followed by 90 minutes at 150 V in 0.1x native cathode buffer. Following electrophoresis, gels were incubated in 150 μM NADH, 3mM nitro blue tetrazolium and 2 mM Tris at pH 7.4 for 3 hours.

Lactate assay

Extracellular lactate was determined using a Lactate-Glo assay kit (Promega, J5022). Media supernatants from $1x10^6$ cells were diluted at 1:50 in PBS and 50 μL were plated in duplicate in a 96-well plate. 50 μL of reaction mix containing lactate dehydrogenase, reductase substrate, NAD, reductase and luciferin detection solution was then added. The resulting luminescence was measured on a BMG microplate reader with an Endpoint of 50 minutes. Lactate levels were assessed using a lactate standard ranging from 1.56 μM to 200 μM. Final results were normalized using each samples protein concentration.

Measurement of itaconate using HPLC

Intracellular itaconic acid levels were measured using high performance liquid chromatography. Pellets from $1x10^6$ macrophages cells were resuspended in PBS, pH 7.4 and lysed by three freeze-thaw cycles. After centrifugation at 17,000 g for 15 minutes at 4°C, debris-free lysates were transferred to new tubes. Proteins were then precipitated following addition of 100 mM of hydrochloric acid and centrifuged at 17,000 g for 15 minutes at 4°C. The remain supernatants were injected onto a column of 250 mm, ACE C-18 column (Hichrom) and itaconate was quantified using UV detection at 210 nm. HPLC separation was performed using a mobile phase comprising of 2.5% acetonitrile and 0.1% phosphoric acid (all ultrapure electrochemical HPLC grade), at a flow rate of 1.0 ml/min. Quantification of itaconic acid was made by comparison with pure itaconic acid (Sigma, I29204) standard range from 0.50 μM to 500 μM. Final results were normalized using each samples protein concentration.

Isocitrate dehydrogenase activity assay

Isocitrate dehydrogenase (IDH) activity was determined for $NADP^+$ dependent isoforms, IDH1 and IDH2, using a colorimetric IDH Assay Kit (Abcam). In duplicate, cell lysates (7.5 μg) and NADPH standards were loaded into 96 well clear plates and incubated with $NADP^+$ and Isocitrate Substrate. NADPH production was recorded on a BMG microplate reader measuring OD_{450nm} in an kinetic assay with an Endpoint of 20 minutes. Endpoint results were used to calculate the amount of NADP in each well from a standard curve, after averaging duplicates and subtracting blank values. IDH activity was determined using the following equation:

$$\text{IDH activity} = (\text{Amount of NADP}/(\text{Time of reaction} \times \text{Volume in well})) \times \text{dilution factor}$$

Aconitase activity assay

The cytosolic aconitase activity was determined using an enzyme activity assay kit (Abcam, ab109712). Cell pellets were lysed into 200 μl of cold assay buffer. After protein concentration determination, 70 μg of each cell lysate was assayed in duplicate into a 96 well UV plate provided and incubated with isocitrate and manganese. The conversion of isocitrate to cis-aconitate was immediately recorded by kinetic measurement at OD_{240nm} for 30 minutes with 60 s intervals and 3 s shaking between readings at room temperature. As recommended by the manual, the resulting aconitase activity was determined by picking two points in a linear increasing interval: Rate (OD/min) = (Absorbance1-Absorbance2) / Time (min).

Measurement of citrate accumulation

Following exposure of macrophages to GSK864, citrate was measured using a Citrate Assay Kit (Sigma) as per manufacturer's instructions.

Cytokine measurements by ELISA

DuoSet enzyme-linked immunosorbent assays (ELISAs) (R&D Systems) were used to measure TNF-α, IL-6, IL-10 and IL-1β in condition media supernatants collected from macrophages re-plated at $1x10^6$ cells per well of a 6 well plate on day 7 of culture and stimulated with LPS/IFNγ for 16 hours. To measure cytokines neat supernatants were used for IL-10 and IL-1β, and supernatants were diluted 1:100 and 1:400 for TNFα and IL-6 respectively to allow comparison with standard curves. The day before the assay Nunc-immuno 96 well plates (ThermoFisher Scientific) were pre-coated overnight at room temperature with Capture antibodies (DuoSet) resuspended in PBS according to the manufacturer's instructions. On the day of the assay plates were washed in Wash Buffer (0.05% Tween20 in PBS) and blocked in Reagent Diluent (1% fatty acid free BSA (Sigma) in PBS) for 1 hour. After further washing, samples and standards were added in duplicate (made up in Reagent Diluent/media as appropriate) and incubated for 2 hours before additional wash steps. Detection antibodies (DuoSet) were added to wells and incubated for 2 hours before additional wash steps. Wells were incubated with Streptavidin-HRP (DuoSet) for 20 minutes protected from light, and then washed again. Substrate Solution (Substrate Reagent Pack, R&D Systems) was added, comprised of 1:1 mixture of Color Reagent A and B, for 20 minutes protected from light. Stop Solution was added (0.67 N H_2SO_4) and plates were read on a BMG microplate reader at 450 and 540 nm. For data analysis, 540 nm readings were subtracted from 450 nm values to correct for optical imperfections in the plates, duplicate readings were averaged and blank optical densities subtracted from standards and samples. Sample concentrations were calculated against standard curves generated using a four-parameter logistic curve-fit, and multiplied by dilution factors if appropriate.

Peritoneal macrophage recruitment

For *in vivo* peritoneal recruitment experiments mice underwent intraperitoneal injected with 4% thioglycolate. 4 days later mice were killed and the peritoneal cavity was lavaged with 5 mL of PBS containing 5 mM EDTA. Macrophages were plated into serological Petri dishes in DMEM/F12 (with 2% FCS, Pen/Strep and Glutamine) and allowed to adhere for 75min. Adherent macrophages were harvested by washing the plates briefly with 2 PBS washes prior to detachment with ice cold PBS/5 mM EDTA then plated for cell activation assays. *In vivo* macrophages were stained with antibodies against CD45 (FITC), CD11b (PerCP) and F4:80 (APC) to assess the purity of the population, specificity of the antibody staining was confirmed using isotype control antibodies stained with the same fluorochromes (all antibodies and isotype controls Biolegend, UK). All flow cytometry was performed using a BD Fortessa X20 cytometer and Diva software (BD Biosciences, Oxford, UK). Data was analyzed using Flow Jo software (TreeStar Inc, Wokingham, UK).

Organomercury enrichment and detection of S-nitrosated IDH by mass spectrometry

BMDMs were isolated, cultured and activated as described above. Phenylmercury resin (kindly provided by Professor Harry Ischiropoulos and Dr Paschalis Doulias, University of Pennsylvania, USA) was used to capture NO-Cys containing proteins, using mass spectrometry as previously described.

Liquid chromatography tandem mass spectrometry (LC-MS/MS) was performed using an Orbitrap Velos mass spectrometer, coupled with a Waters nanoAquity UPLC. In brief, injected samples underwent online desalting using a Trap column Symmetry C18, 180 μm × 20 mm, 5 μm particle, Waters). For separation, a BEH C18 column (75 μm × 250 mm, 1.7 μm particle, Waters) was used with a flow rate of 250 nl/min over 60 minutes and a gradient of 3%–40% acetonitrile 0.1% Formic acid. Survey scans were acquired in the orbitrap with a resolution of 60,000 at 400 m/z between 300 and 2000 m/z for up to 100 ms with an ion target of 1E6. Selected precursors were picked above a threshold of 5E2 counts for MS/MS and excluded for 30 s after sampling. MS/MS spectra were acquired in the ion trap with a maximum accumulation time of 100 ms and an ion target of 5E4 counts. CID fragmentation was performed with a normalized collision energy of 35. Experiments were conducted in biological triplicates and all sample analyzed in a single sample batch.

QUANTIFICATION AND STATISTICAL ANALYSIS

Statistical analysis

All statistical analyses were carried out using Microsoft Excel (Microsoft) and GraphPad Prism 7 (GraphPad Inc.) software. Data were expressed as mean ± standard error of the mean (SEM). A Student's t test was used to compare two groups affected by one single variable. Two-way ANOVA was used to compare multiple data groups affected by two independent variables, with Tukey's post-test to compare groups with each other. Differences were considered statistically significant at *P values* of * $p < 0.05$.

Proteomic data analysis

The data was analyzed in R using the default "stats" package for Analysis of Variance (ANOVA) and Tukey's 'Honest Significant Different' (TukeyHSD) package for post hoc test on all the proteins. The data comprised of 5,704 different proteins measured on primary macrophages (n = 4) from $Gch^{fl/fl}$ and $Gch^{fl/fl}$Tie2cre mice that were unstimulated or harvested following activation with LPS/IFNγ. We performed a two-way ANOVA comparing a panel of proteins in $Gch^{fl/fl}$Tie2cre against the $Gch^{fl/fl}$ samples followed by TukeyHSD method to create a set of confidence intervals based on the sample means. The proteins having a *P*-value $< 9x10^{-6}$ (based on Bonferroni correction = 0.05/5704) were considered significant.

Enrichment analysis was carried out using the ClueGO app in Cytoscape (Ref), by searching gene names encoding proteins that were considered statistically significant by and changed abundance ≥ 20%, against 4 data bases: Gene ontology (GO) Biological Process (GO-BP), GO Cellular Component (GO-CC), GO Molecular Function (GO-MF) and Kyoto Encyclopaedia of Genes and Genomes (KEGG) pathways. Significantly enriched terms ($p < 0.05$) containing ≥ 3 genes covering ≥ 4% of genes in that term were used to create ClueGO Layouts, where each node represents a term (GO-BP, -CC and -MF are circular and KEGG are hexagonal) and the size depends on the *P value* (smaller p = bigger node). GO term fusion was applied to merge nodes containing highly similar genes, and terms were grouped into networks based on their kappa score level (≥0.3). Nodes of the same color have overlapping functional networks and the most significant term in each group was labeled with all others hidden for simplicity. All node/term information was downloaded together with names of the genes identified in our dataset that lead to enrichment, and these were used to create heatmaps showing the scaled abundance of proteins encoded by these genes using Prism 7.

Metabolomic data analysis

Bioinformatics: The informatics system consisted of four major components, the Laboratory Information Management System (LIMS), the data extraction and peak-identification software, data processing tools for QC and compound identification, and a collection of information interpretation and visualization tools for use by data analysts. The hardware and software foundations for these informatics components were the LAN backbone, and a database server running Oracle 10.2.0.1 Enterprise Edition.

DATA AND CODE AVAILABILITY

Proteomics dataset is available on the PRIDE proteomics data repository: database accession number: PRIDE: PXD010628.

Cell Reports, Volume *28*

Supplemental Information

Nitric Oxide Modulates Metabolic Remodeling in Inflammatory Macrophages through TCA Cycle Regulation and Itaconate Accumulation

Jade D. Bailey, Marina Diotallevi, Thomas Nicol, Eileen McNeill, Andrew Shaw, Surawee Chuaiphichai, Ashley Hale, Anna Starr, Manasi Nandi, Elena Stylianou, Helen McShane, Simon Davis, Roman Fischer, Benedikt M. Kessler, James McCullagh, Keith M. Channon, and Mark J. Crabtree

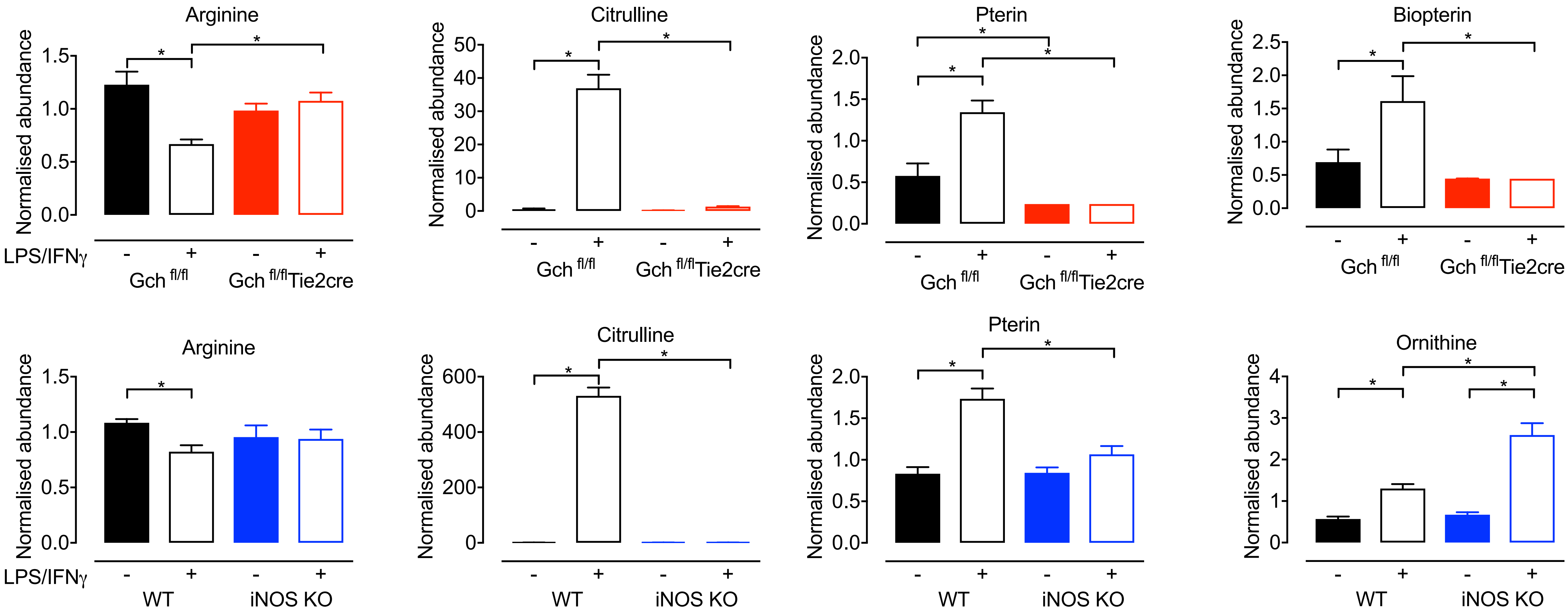

Supplemental figure 1. Quantification of BH4 and NOS related pathway metabolites. Related to Figure 1. Metabolomic measurements of Arginine, Citrulline, Pterin and Biopterin (n=6) Data are mean + SEM. *P* values calculated using 2-way ANOVA with Tukey's post-test (**P*<0.05).

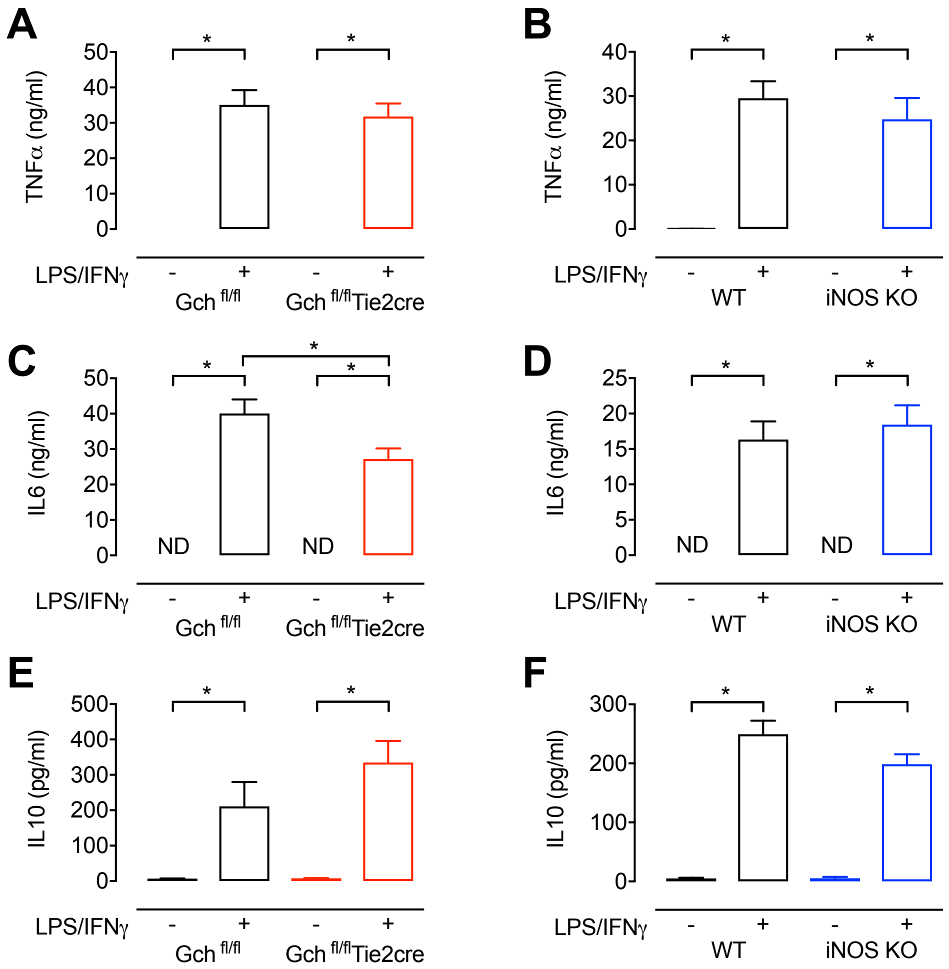

Supplemental figure 2. Inflammatory cytokines are induced in NO-deficient cells. Related to Figure 1. ELISA measurements of TNFα in (A) Gch$^{fl/fl}$Tie2cre and (B) iNOS KO cells, IL-6 in (C) Gch$^{fl/fl}$Tie2cre and (D) iNOS KO cells and IL-10 in (E) Gch$^{fl/fl}$Tie2cre and (F) iNOS KO cells. (n=5) Data are mean + SEM. *P* values calculated using 2-way ANOVA with Tukey's post-test (**P*<0.05).

A

Unstimulated Gch$^{fl/fl}$ Tie2cre vs Gch$^{fl/fl}$

Protein	Fold Change
Squalene synthase	2.69
Adenylate kinase 4, mitochondrial	2.25
Thioredoxin interacting protein	1.59
C5a anaphylatoxin chemotactic receptor 1	1.54
Extracellular serine/threonine protein kinase FAM20C	1.49
PTB-containing, cubilin and LRP1-interacting protein	1.42
Beta-1,4 N-acetylgalactosaminyltransferase 1	1.41
6-phosphofructokinase, liver type	1.37
Class II histocompatibility antigen, M alpha chain	-1.36
C-type lectin domain family 6 member A	-1.36
Urokinase-type plasminogen activator	-1.38
MCG21506	-1.41
Interferon regulatory factor 4	-1.41
Methylcytosine dioxygenase TET2	-1.43
Catenin beta-1	-1.64
Peroxisomal acyl-coenzyme A oxidase 3	-1.93
Placenta-expressed transcript 1 protein	-2.22
Cytochrome P450 2S1	-2.39
Arsenite methyltransferase	-3.76
GTP cyclohydrolase 1 (GTPCH)	-4.40

B

LPS/IFNγ treated Gch$^{fl/fl}$ Tie2cre vs Gch$^{fl/fl}$

Protein	Fold Change
R3H and coiled-coil domain-containing protein 1	4.95
Protein Ms4a7	2.90
Serum amyloid A-3 protein	2.30
NADH dehydrogenase [ubiquinone] 1 alpha subcomplex subunit 12 (NDUFS12)	1.96
Fatty acid desaturase 2	1.89
Protein Gm20547	1.79
Cytochrome P450 4V2	1.77
NADH dehydrogenase [ubiquinone] iron-sulfur protein 6, mitochondrial (NDUFS6)	1.73
Radical S-adenosyl methionine domain-containing protein 2	-1.84
Peroxisomal acyl-coenzyme A oxidase 3	-1.87
DEP domain-containing mTOR-interacting protein	-1.89
Solute carrier family 2, facilitated glucose transporter member 1 (GLUT-1)	-1.97
Platelet-activating factor acetylhydrolase	-1.99
OX-2 membrane glycoprotein	-2.15
Cytochrome P450 2S1	-2.16
Stearoyl-CoA desaturase-3	-2.42
BCL2/adenovirus E1B 19 kDa protein-interacting protein 3	-2.84
Arsenite methyltransferase	-3.07
Isoform 2 of sphingosine kinase 1	-4.67
GTP cyclohydrolase 1 (GTPCH)	-8.96

C

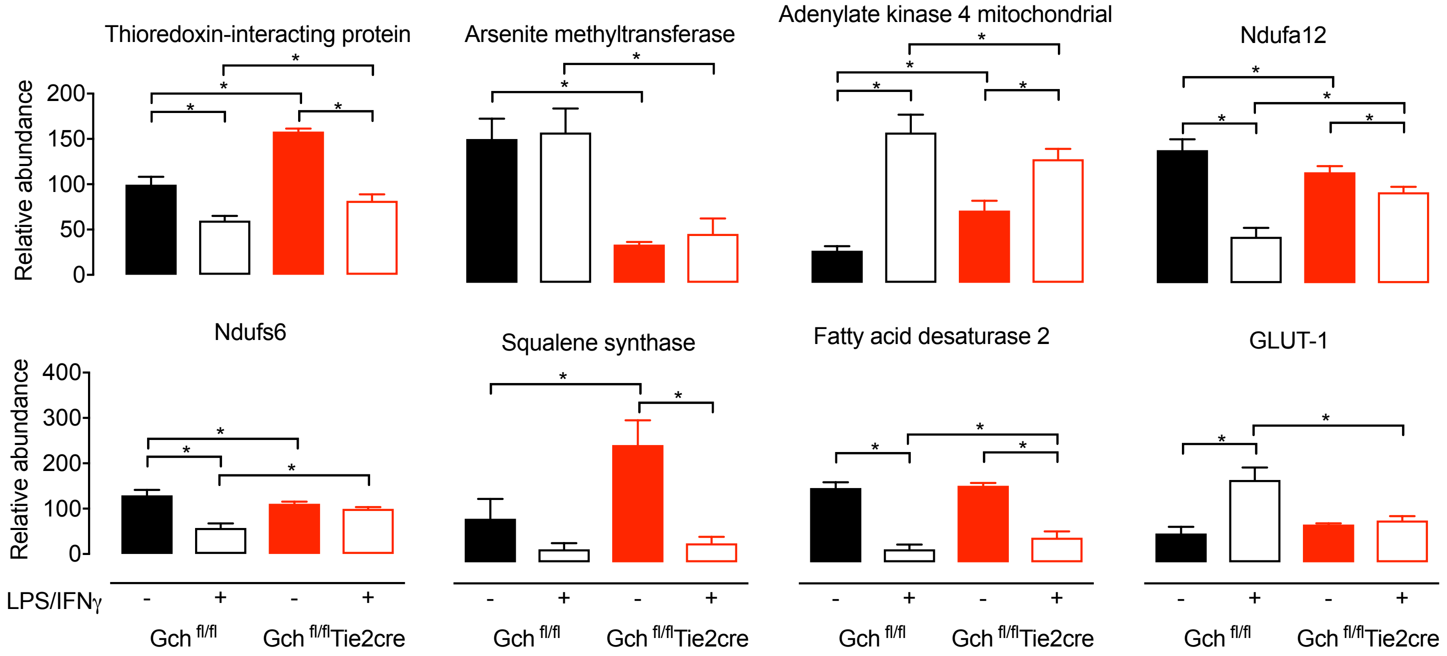

Supplemental figure 3. The proteins with the most changed abundance in Gch$^{fl/fl}$Tie2cre vs Gch$^{fl/fl}$ macrophages. Related to Figure 1. (A-B) The 20 proteins with the most changed abundance in Gch$^{fl/fl}$Tie2cre vs Gch$^{fl/fl}$ macrophages (A) unstimulated or (B) stimulated with LPS and IFNγ. (C) Abundance of selected proteins from A and B. (n=4) Data are mean + SEM (**P*<0.05).

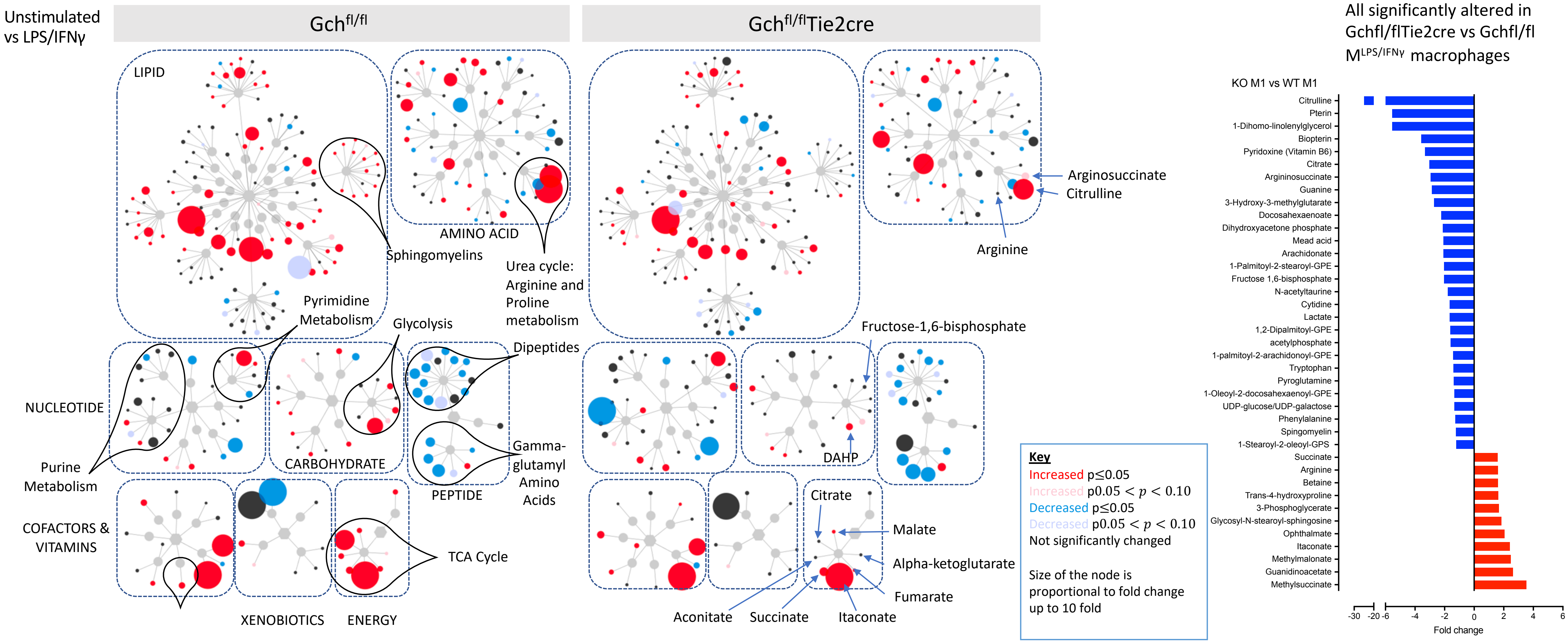

Supplemental figure 4. Cytoscape analysis of metabolomic data from Gch$^{fl/fl}$Tie2cre vs Gch$^{fl/fl}$ macrophages. Related to Figure 4 and 5.

(n=6) Data are mean + SEM. *P* values calculated using 2-way ANOVA (**P*<0.05).

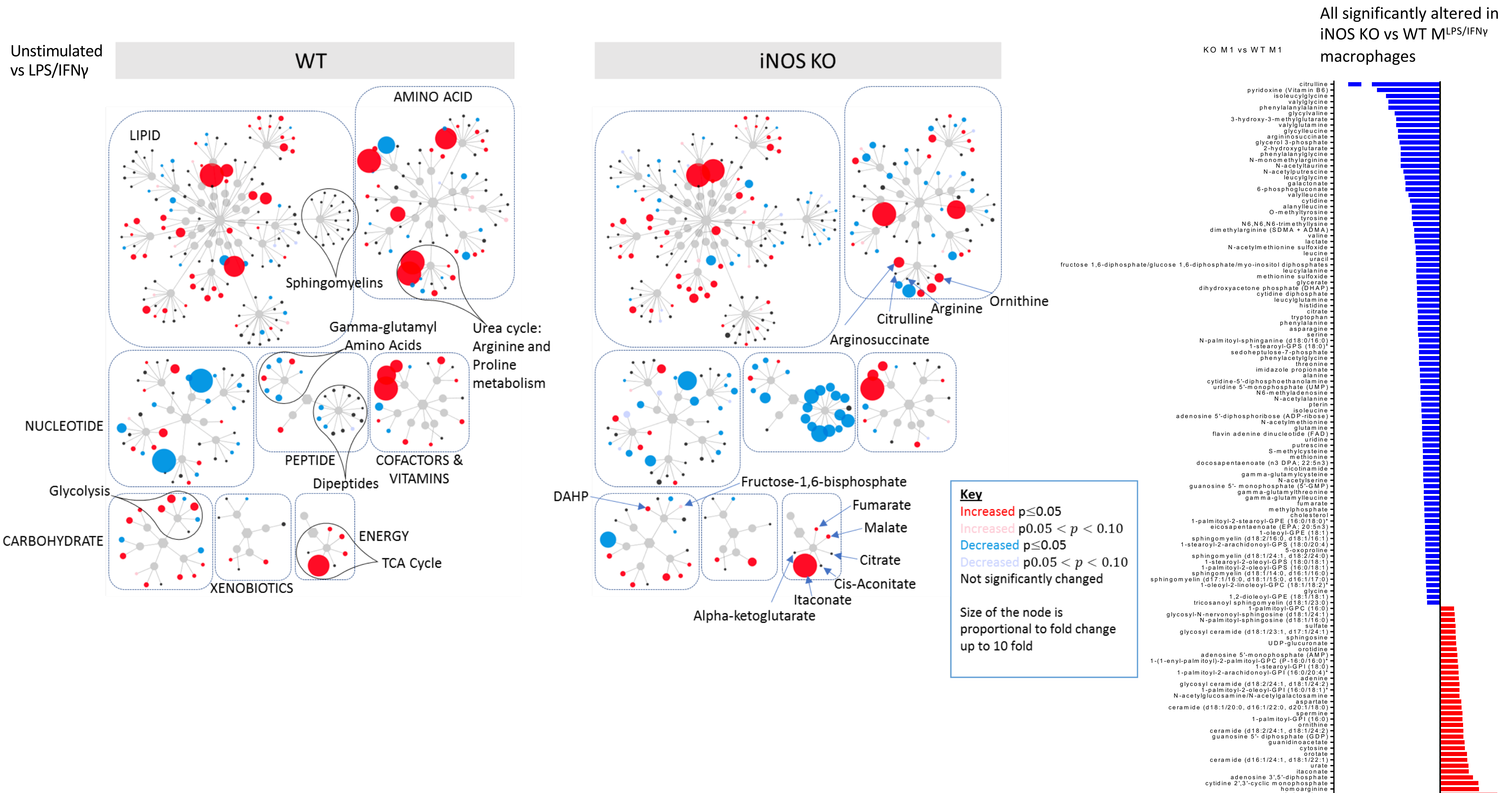

Supplemental figure 5. Cytoscape analysis of metabolomic data from iNOS KO vs WT macrophages. Related to Figure 4 and 5. (n=6) Data are mean + SEM. *P* values calculated using 2-way ANOVA (**P*<0.05).

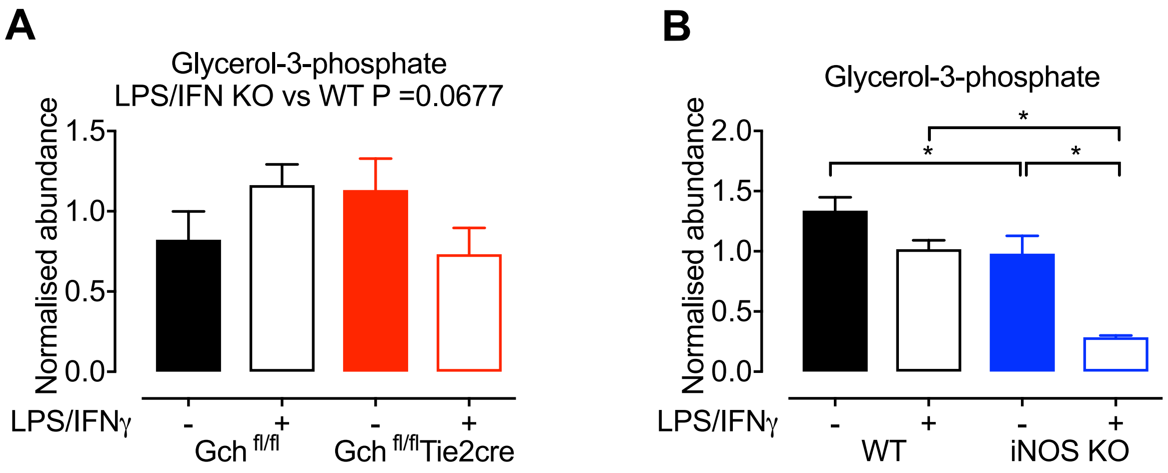

Supplemental figure 6. Glycerol-3-phosphate is decreased in NO deficient activated macrophages. Related to Figure 4. (A-B) Glycerol-3-phosphate abundance measured by metabolomics. (n=6) Data are mean + SEM. *P* values calculated using 2-way ANOVA (**P*<0.05).

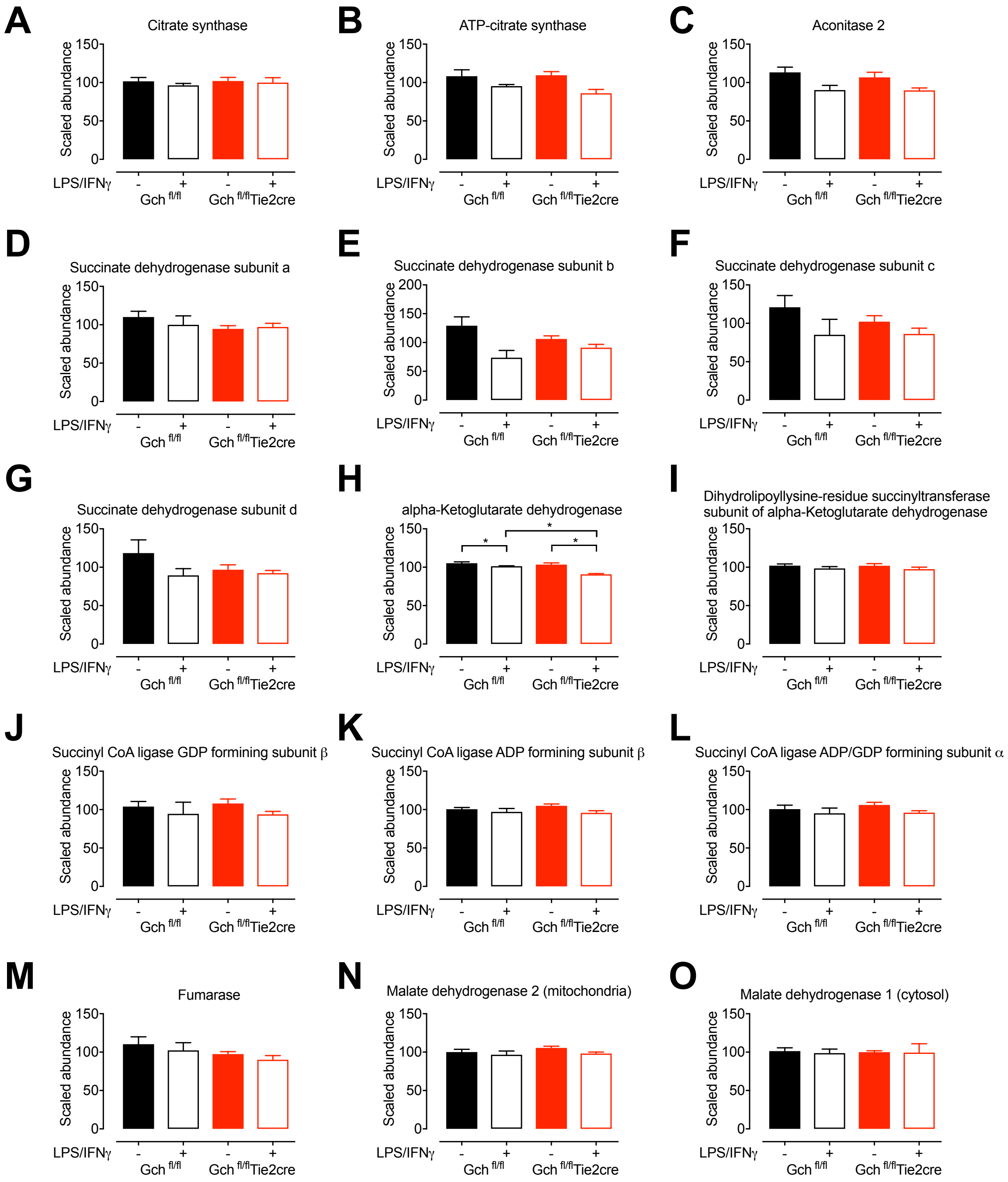

Supplemental figure 7. The abundance of TCA cycle enzymes, measured by metabolomics. Related to Figure 5. (n=6).

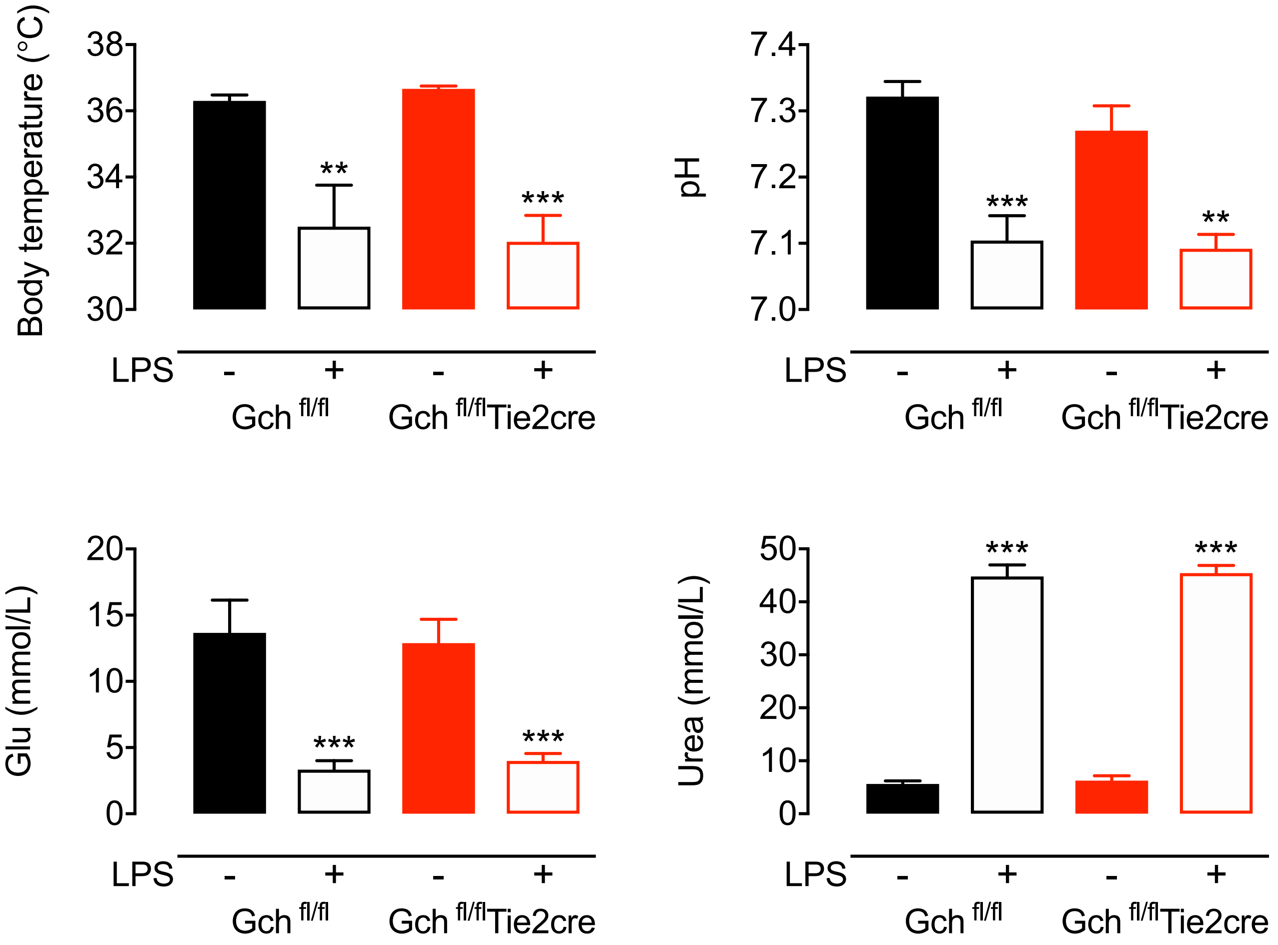

Supplemental figure 8. Physiological responses to acute endotoxaemia, induced by LPS (12.5 mg/kg). Related to Figure 7.

LPS/IFNγ	$Gch^{fl/fl}$		$Gch^{fl/fl}$Tie2cre	
	-	+	-	+
Citrate	0.378 ± 0.158	2.745 ± 0.337	0.804 ± 0.275	0.915 ± 0.238
cis-Aconitate	0.845 ± 0.368	1.770 ± 0.293	1.278 ± 0.378	1.079 ± 0.238
alpha-Ketoglutarate	0.788 ± 0.084	0.973 ± 0.116	1.223 ± 0.163	1.369 ± 0.155
Succinate	0.521 ± 0.080	1.339 ± 0.223	0.677 ± 0.124	2.122 ± 0.193
Fumarate	0.563 ± 0.148	1.160 ± 0.245	1.115 ± 0.283	1.445 ± 0.180
Malate	0.597 ± 0.130	1.132 ± 0.141	0.942 ± 0.183	1.271 ± 0.106
Itaconate	0.118 ± 0.010	2.390 ± 0.483	0.272 ± 080	5.780 ± 0.315

LPS/IFNγ	WT		iNOS KO	
	-	+	-	+
Citrate	0.873 ± 0.069	1.750 ± 0.147	0.865 ± 0.085	0.896 ± 0.098
cis-Aconitate	0.951 ± 0.074	1.095 ± 0.032	0.952 ± 0.078	1.051 ± 0.125
alpha-Ketoglutarate	0.871 ± 0.132	0.902 ± 0.144	0.914 ± 0.066	0.853 ± 0.144
Succinate	ND	ND	ND	ND
Fumarate	0.892 ± 0.090	1.685 ± 0.182	0.781 ± 0.073	1.200 ± 0.108
Malate	0.806 ± 0.082	1.779 ± 0.0260	0.731 ± 0.054	1.324 ± 0.135
Itaconate	0.192 ± 0.049	1.738 ± 0.139	0.234 ± 0.068	4.362 ± 0.232

Supplemental table 3. Metabolite abundances presented in heat map from Figure 5. Metabolite abundance measured by metabolomics. (n=6) Data are mean + SEM.

Proteins encoded by genes induced by HIF1α	References
GLUT-1	(Chen et al., 2001; Liu et al., 2016; Wang et al., 2017)
Hexokinase-1	(Luo et al., 2006)
Hexokinase-2	(Mathupala et al., 2001; Riddle et al., 2000)
PFKFB3	(Liu et al., 2016; Obach et al., 2004)
Phosphoglucomutase-1	(Hu et al., 2003; Pelletier et al., 2012)
Glycogen synthase	(Pescador et al., 2010)

Supplemental table 4. Glycolytic and glycogen metabolising proteins encoded by genes regulated by HIF1α. Related to Figures 4 and 5.
